# Physiological and molecular mechanisms of exogenous salicylic acid in enhancing salt tolerance in tobacco seedlings by regulating antioxidant defence system and gene expression

**DOI:** 10.3389/fpls.2025.1545865

**Published:** 2025-01-31

**Authors:** Xiliang Song, Jian Chen, Can Xu, Xianjie Cai, Wenjing Song, Aixia Chang, Yu Zhang, Chenggang Luo

**Affiliations:** ^1^ College of Life Sciences, Dezhou University, Dezhou, China; ^2^ Shanghai Tobacco Group Co. Ltd, Shanghai, China; ^3^ Tobacco Research Institute of Chinese Academy of Agricultural Sciences China, Qingdao, China

**Keywords:** soil salinity, iron toxicity, oxidative damage, salicylic acid, salt tolerance

## Abstract

**Introduction:**

Salt stress has emerged as a predominant abiotic factor that jeopardizes global crop growth and yield. The plant hormone salicylic acid (SA) has notable potential in mitigating salt toxicity, yet its mechanism in enhancing the salinity tolerance of tobacco plants is not well explored.

**Methods:**

This study aimed to assess the potential benefits of exogenous SA application (1.0 mM) on tobacco seedlings subjected to saline soil conditions.

**Results:**

The foliar spray of SA partially mitigated these salt-induced effects, as evidenced by a reduction of malondialdehyde content, and improvements of leaf K^+^/Na^+^ ratios, pigment biosynthesis, and electron transport efficiency under NaCl stress. Additionally, SA increased the contents of total phenolic compound and soluble protein by 16.2% and 28.7% to alleviate NaCl-induced oxidative damage. Under salt stressed conditions, the activities of antioxidant enzymes, including superoxide dismutase, ascorbate peroxidase, catalase, and peroxidase increased by 4.2%~14.4% in SA sprayed tobacco seedlings. Exogenous SA also increased ascorbate and glutathione levels and reduced their reduced forms by increasing the activities of glutathione reductase, ascorbate peroxidase, monodehydroascorbate reductase and dehydroascorbate reductase. qRT−PCR analysis revealed that the key genes regulating SA biosynthesis, carbon assimilation, the antioxidant system and the ascorbate−glutathione cycle were activated by SA under conditions of salt stress.

**Discussion:**

Our study elucidates the physiological and molecular mechanisms of exogenous SA in enhancing plant salt tolerance and provides a practical basis for crop improvement in saline environments.

## 1 Introduction

Soil salinity is acknowledged as a significant abiotic stressor that severely jeopardizes food security by negatively affecting agricultural productivity (Liu et al., 2024; Mahawar et al., 2024; Xiao and Zhou, 2023). Over 9% of the global arable land is under saline-alkali stress, with a particular impact on arid and semiarid areas of the world (FAO, 2024). The excess uptake of soluble salt ions, especially Na^+^ and Cl^-^, disrupts the K^+^/Na^+^ ratio in plant tissues, leading to osmotic stress, ionic toxicity, and nutritional imbalances in plants (Isayenkov and Maathuis, 2019). Salt stress also induces the production of reactive oxygen species (ROS) (Pan et al., 2021), causing oxidative stress and cellular damage (Sheikhalipour et al., 2021), which in turn seriously affects plant growth and crop yield (Kumar et al., 2022). Addressing or mitigating the detrimental impacts of salinity on plants is an imperative challenge for sustainable agricultural yields.

To mitigate the excess generation of ROS and preserve redox balance, plants have evolved a suite of adaptive mechanisms to bolster their resilience against salt stress. These strategies include the regulation of osmotic pressure, maintenance of mineral homeostasis, activation of antioxidant defences, production of hormones, generation of nitric oxide, and synthesis of osmoprotectants (Isayenkov and Maathuis, 2019; Keisham et al., 2018; Ramasamy and Mahawar, 2023). Despite these intrinsic protective measures, these mechanisms provide only partial protection against the adverse impacts of salt stress. Over the past few years, notable progress has been made in alleviating salt-induced toxicity and enhancing plant tolerance to saline via the application of detoxifying antioxidants, including proline (Koc et al., 2024), nitric oxide (Wang et al., 2024), jasmonic acid (Ali et al., 2022), salicylic acid (SA) (Kumar et al., 2024), and melatonin (Kaya et al., 2022). Among these protective substances, SA stands out as a natural phenolic endogenous signalling molecule that plays important roles in modulating the antioxidant defence cascade and ROS scavenging to help plants acclimatize to adverse environments, such as heavy metal toxicity (Maghsoudi et al., 2020), water deficit (Kareem et al., 2019), salinity (Yadav et al., 2020), heat stress (Arif et al., 2020), and pathogenic infections (Zulfiqar and Ashraf, 2021). In recent years, there has been a growing focus on the role of SA in enhancing plant tolerance to saline conditions (Batista et al., 2019; Barwal et al., 2024). For example, foliar spray of SA at 1.0 mL increased leaf K^+^ and Ca^2+^ contents, stimulated glycine betaine production, and improved the enzymatic activities of peroxidase (POX), catalase (CAT), and superoxide dismutase (SOD), leading to a considerable increase in the crop yield of soybean plants grown in 10 dS m^-1^ NaCl (Farhangi-Abriz and Ghassemi-Golezani, 2018). Another study reported that exogenous application of 0.3 mM SA alleviated the negative impacts of salt stress on cucumber seedlings by promoting leaf photosynthesis, modulating the transcription levels of genes involved in root system architecture, and increasing the number and length of lateral roots (Miao et al., 2020). The investigation of the impact of SA on crop plants and its potential role in defence against diverse stressors can significantly contribute to bridging the gap in effective strategies for crop improvement under saline conditions.

Studies have revealed that SA acts as an important plant growth regulator and plays pleiotropic roles in mitigating salinity toxicity at the physiological and molecular levels. Under salinity stress conditions, SA-mediated mechanisms of salinity tolerance contribute to (a) increasing the expression of genes related to the ascorbate-glutathione (AsA-GSH) cycle, such as monodehydroascorbate reductase (MDHAR), glutathione reductase (GR), dehydroascorbate reductase (DHAR), and glutathione peroxidase (GPX) (Zheng et al., 2018; Liu et al., 2024), as well as enzymes that metabolize hydrogen peroxide (H_2_O_2_), including CAT, POX, SOD, and glutathione-S-transferase (Ahanger et al., 2020; Barwal et al., 2024); (b) increasing the biosynthesis of key plant growth hormones, namely, auxins, gibberellins, and abscisic acid (Shakirova et al., 2003; Sharma et al., 2020; Kashif et al., 2024), which play an integral role in facilitating plant adaptation to saline conditions; (c) increasing the concentration of antioxidants, including proline, glycine betaine, flavonoids, and alkaloids, which are essential for combating oxidative stress induced by salt stress (Alizadeh et al., 2024; Barwal et al., 2023; Farhangi-Abriz and Ghassemi-Golezani, 2018); and (d) improving leaf photosynthetic ability by restoring photosystems, improving leaf pigments, modulating plant carbohydrate metabolism, and sustaining chloroplast membrane integrity, which is vital for energy production and stress tolerance (Islam et al., 2023; Lotfi et al., 2020; Ma et al., 2017). Furthermore, exogenous SA can effectively modulate the Na^+^ and K^+^ balance in the cytoplasm by inhibiting K^+^ leakage, regulating GORK channels, increasing H+-ATPase activity, and providing the necessary energy for the K^+^/Na^+^ exchanger on the plasma membrane, thereby facilitating plant adaptation to saline conditions (Kumar et al., 2024; Jayakannan et al., 2015). SA also increases metabolite production, which aids in compartmentalizing Na^+^ and K^+^ between roots and shoots, thereby reducing the effects of salt stress on photosynthesis by influencing primary and volatile organic compounds (Batista et al., 2019).

Numerous studies have investigated the role of SA in diminishing the adverse effects of salt stress across a variety of plant species, such as wheat (Hafeez et al., 2024), soybean (Farhangi-Abriz and Ghassemi-Golezani, 2018), and *Egletes viscosa* (Batista et al., 2019). However, the specific mechanisms by which this growth regulator improves plant salt stress tolerance are still not fully understood. Therefore, the current study aimed to assess the effects of the foliar application of salicylic acid (SA) on mitigating the oxidative, ionic, and osmotic stresses associated with salt toxicity in tobacco, a well-established model plant for this type of research. We hypothesize that exogenous SA effectively defends against salt toxicity and scavenges ROS by stimulating enzymatic and nonenzymatic defence mechanisms, which subsequently increases the photosynthetic performance and growth of tobacco plants. The primary objective of this study was to clarify the impact of SA application on alleviating the negative effects of salinity stress in tobacco, with a focus on both physiological and molecular aspects. Special attention has been given to understanding how SA, as a biostimulant, modulates the ASA−GSH pathway. The outcomes of this research are expected to yield an effective ecological and economical strategy for crop cultivation in saline environments.

## 2 Materials and methods

### 2.1 Plant growth and treatment conditions

The seeds of tobacco (c.v. Zhongchuan 208) were graciously supplied by the Chinese Academy of Agricultural Sciences. After sterilization with 1% KMnO_4_ for 15 min and six rinses with sterile distilled water, the seeds were planted in plastic pots containing perlite and peat moss (1:1 v/v) to facilitate germination in an artificial climate chamber. The growth conditions were controlled as follows: photosynthetically active radiation of 900 µmol m^-2^ s^-1^, a day/night temperature of 28/25°C, and an air humidity of 70%. When the third true leaf emerged, the seedlings were transferred to 3.0 L pots containing coarse sand. The pots were filled with Hoagland’s nutrient mixture to supply macro- and microelements, and the pH was maintained at 6.7–7.2. The Hoagland nutrient mixture was replenished every two days.

The four treatments included CK (0.0 mM NaCl+0.0 mM SA), NaCl (200.0 mM NaCl+0.0 mM SA), CK+SA (0.0 mM NaCl + 1.0 mM SA), and NaCl+SA (200.0 mM NaCl+1.0 mM SA), with quadruplicate replicates. An aqueous solution of SA prepared in Milli-Q water was sprayed onto the leaf surface every day. The treatments were laid out in a completely randomized design. After a 15-day treatment period, tobacco plants were sampled to assess the impact of NaCl and SA on growth, physiological parameters, and the expression of target genes.

### 2.2 Chlorophyll fluorescence and photosynthetic pigments

The second apical tobacco leaves were selected for leaf OJIP transient measurements. After 20 min of dark acclimation, the chlorophyll (Chl) a fluorescence induction transients were measured with an M-PEA Analyser (Hansatech Ltd., Norfolk, UK). The Chl fluorescence parameters include the minimal fluorescence (Fo), maximal fluorescence (Fm), activity of the water-splitting complex (Fv/Fo), performance index (PI_ABS_), energy flux rate (Mo), light absorption energy flux per RC (ABS/RC), relative energy flux per PSII RC (DIo/RC), maximum electron transport flux per PSII RC (ETo/RC), trapped energy flux per R C (TRo/RC), maximum quantum yield for primary photochemistry (φ_Po_), quantum yield for electron transport (φ_EO_), probability that an electron moves further than QA (ψ_o_), relative variable fluorescence at the I-step (Vi), relative variable fluorescence at the J-step (Vj), normalized total complementary area above the OJIP transient (S_M_), and number of QA redox turnovers until Fm is reached (N), accompanied by their formulas as listed in our previous study (Li et al., 2020).

After the measurements of the leaf OJIP transient were completed, the leaves were sampled to measure the leaf pigment contents. Briefly, a homogenate of 0.1 g of fresh leaves was prepared using 80% acetone. The resulting mixture was centrifuged, and the supernatant was carefully collected. Following the methodology of Song et al. (2022), absorbance measurements were conducted at specific wavelengths of 663, 646 and 470 nm to calculate the contents of leaf Chl a, Chl b, and carotenoids (Cars), respectively.

### 2.3 Leaf cation contents

Approximately 1.0 g of desiccated tobacco foliage was used for the determination of Na^+^ and K^+^ ion concentrations. The leaves were desiccated at 550°C for 7 hours in an electric furnace. The resulting dry material was subsequently pulverized and digested in 5 M HNO_3_ at a controlled temperature of 25°C for 24 hours. Following digestion, the samples were transferred to a hot plate maintained at 120°C and incubated for an additional hour. The digested samples were then diluted with 50 mL of double-distilled water to facilitate analysis using an atomic absorption spectrophotometer (PerkinElmer, NexION 5000G, USA).

### 2.4 Total soluble protein contents

For the quantification of total soluble protein (TSP) content, 0.5 g of each leaf sample was homogenized with 6.25 mL of Tris-HCl buffer (pH 7.5) and then centrifuged at 16,000 rpm for 30 minutes. Reagents were prepared as follows: Reagent A contains Na_2_CO_3_, 0.5 N NaOH, 1% CuSO_4_, and 2% sodium potassium tartrate. Reagent B contains Folin−Ciocalteu reagent. To the supernatant, 1 mL of Reagent A and 3 mL of Reagent B were added, followed by incubation at 15°C. The absorbance of the resulting solution was measured at 625 nm to determine the TSP content following the method of Lowry et al. (1951).

### 2.5 Total phenolic compounds

The leaf total phenolic (TPC) content was assessed using the Folin−Ciocalteu assay as outlined by Kähkönen et al. (1999). For this purpose, 0.5 g of leaf material was immersed in 10 mL of methanol for extraction. Then, 40 μL of the resulting extract, adjusted to 80% methanol concentration, or an equivalent volume of a gallic acid calibration solution, was combined with 1.8 mL of the Folin−Ciocalteu reagent that had been diluted 10 times with distilled water. The resulting mixture was incubated at ambient temperature for 5 minutes, after which 1.2 mL of a 7.5% (w/v) sodium carbonate solution was added. Following a 60-minute incubation at room temperature, the absorbance at 765 nm was recorded.

### 2.6 Malondialdehyde content

The malondialdehyde (MDA) content was assayed using the methods of Dhindsa et al. (1981). Initially, 0.5 g of leaf material was ground with 5 mL of a 0.1% (w/v) solution of trichloroacetic acid (TCA). The homogenate was then spun down at 12,500×g for 20 minutes at 25°C to yield a supernatant. Two millilitres of this supernatant was added to 2 mL of a newly prepared solution containing both thiobarbituric acid (TBA) and TCA. The combined mixture was heated at 90°C for 30 minutes to develop the chromogen, and the reaction was stopped by placing the tubes in an ice bath for 10 minutes. The absorbance of the chromogen was recorded at 520 nm and 600 nm to determine the MDA levels.

### 2.7 Ascorbate and glutathione contents

A fresh leaf sample weighing 0.5 g was homogenized in 3 mL of a cold solution containing 5% meta-phosphoric acid and 1 M EDTA. After centrifugation at 11,500×g, the supernatant was collected for the analysis of glutathione (GSH) and ascorbate (AsA), following the protocol established by Huang et al. (2005). A 0.4 mL sample of the supernatant was neutralized with 0.6 mL of 500 mM K-phosphate buffer (pH 7.0) and incubated with 100 mM K-phosphate buffer (pH 7.0) containing 0.5 units of ascorbate oxidase. The absorbance at 265 nm was recorded to determine the amount of reduced AsA. To measure total AsA, the supernatant was treated with 30 mM dithiothreitol, and the absorbance was measured at 265 nm. The dehydroascorbate (DHA) concentration was calculated as the difference between the total AsA concentration and the reduced AsA concentration. For the reduced GSH and oxidized glutathione (GSSG) assays, the method described by Yu et al. (2003) was used. A 0.4 mL sample was neutralized with 0.6 mL of 500 mM K-phosphate buffer (pH 7.0). The GSH content was assessed by monitoring the absorbance at 412 nm due to the formation of 2-nitro-5-thiobenzoic acid from the reduction of 5,5′-dithio-bis(2-nitrobenzoic acid). GSSG was quantified after the removal of GSH using 2-vinylpyridine as a derivatizing agent.

### 2.8 Antioxidant enzyme activities

Newly picked leaf material was ground in chilled potassium phosphate buffer (50 mM, pH 7.8). The resulting suspension was subjected to centrifugation at 4°C with a rotational speed of 10,000 rpm for 20 minutes. The supernatant was extracted for enzyme activity measurements. Superoxide dismutase (SOD) activity was evaluated using the nitro blue tetrazolium (NBT) photoreduction technique as reported by Giannopolitis and Ries (1977). This approach gauges SOD activity based on the extent of inhibition of NBT photoreduction in the presence of the enzyme. For the measurement of peroxidase (POD) activity, the guaiacol method outlined by Zhang and Kirkham (1994) was utilized. This involves observing the increase in absorbance at 470 nm due to the oxidation of guaiacol by POD. Catalase (CAT) activity was determined via the ammonium molybdate method as described by Pomeroy et al. (1975), where CAT activity is inferred from the decrease in absorbance at 240 nm, indicative of hydrogen peroxide (H_2_O_2_) decomposition. Ascorbate peroxidase (APX) activity was assayed by monitoring the oxidation of ascorbate as detailed by Nakano and Asada (1987), which is quantified by the decrease in absorbance at 290 nm due to ascorbate oxidation by APX.

Glutathione reductase (GR) activity was evaluated using the technique described by Foyer and Halliwell (1976), which involves measuring the decrease in absorbance at 340 nm. The assay mixture was composed of Tris-HCl buffer (100 mM, pH 8.0), EDTA (0.5 mM), MgCl2 (0.5 mM), GSSG (10 mM), NADPH (1 mM), and the enzyme extract. Monodehydroascorbate reductase (MDHAR) activity was determined in accordance with the method of Miyake and Asada (1992), and the reduction in optical density at 340 nm was monitored. The assay mixture contained HEPES-KOH buffer (50 mM, pH 7.6), AsA oxidase (2.5 units), NADH (1.0 mM), AsA (2.5 mM), and the enzyme extract. Dehydroascorbate reductase (DHAR) activity was assayed following the protocol of Nakano and Asada (1981), which tracks the reduction in optical density at 265 nm. The reaction mixture for this assay included HEPES-KOH buffer (100 mM, pH 7.0), GSH (20 mM), and DHA (2 mM).

### 2.9 Plant biomass

The collected plants were meticulously divided into two sections: the aerial parts (shoots) and the underground parts (roots). Each section was washed extensively with sterile distilled water three times to remove any surface impurities. After washing, the plant tissues were dried in an oven at 105°C for 20 minutes to remove any surface moisture and then dried further at 70°C until they reached a stable weight. The dry weights of the shoot biomass (SB) and root biomass (RB) were recorded. The total biomass (GB) was computed by summing the SB and RB, and the root-to-shoot ratio (R/S) was ascertained by dividing the RB by the SB.

### 2.10 Quantitative real-time PCR analysis

Complementary DNA (cDNA) was generated via the PrimeScript™ RT Reagent Kit from TaKaRa, Japan, in accordance with the manufacturer’s guidelines. Quantitative real-time PCR (qRT−PCR) was performed using random hexamer primers. The target gene-specific primers were designed via Primer Premier v5.0 software from Premier Biosoft, USA, and their selectivity was verified via BLASTN database searches. Prior to qRT−PCR, a melting curve analysis was conducted for each primer pair to ensure the specificity of the amplification. The qRT−PCRs were performed in a 20 μL volume, with TransStart^®^ Top Green qPCR SuperMix provided by TransGen Biotech, China, adhering to the manufacturer’s protocol. The CFX96™ Real-Time System from Bio-Rad, USA, was used to measure fluorescence intensity. Each PCR was repeated four times to ensure the reliability of the results. To standardize the expression data of the target genes, the *actin* gene, a housekeeping gene with consistent expression, was used as an internal control. The relative transcript levels were determined using the 2^-ΔΔCt^ method. Each gene per leaf sample was tested in quadruplicate for both biological and technical replications. Additional details about the PCR primers are presented in **Supplementary Table S1**.

### 2.11 Statistical analysis

Data analysis was conducted via SPSS version 24.0 (SPSS Inc., USA), with one-way ANOVA used to identify significant differences across treatment groups. All data including plant physio-biochemical parameters and gene relative expression levels are presented as the means ± standard errors (SE), which were calculated from four distinct replications. For further analysis, *post hoc* tests, specifically Duncan’s multiple range test, were implemented to identify significant differences at the *p* < 0.05 threshold. Correlation assessments were executed with the ‘corrplot’ package in R version 4.4.1. SmartPLS 3.0 was employed for structural equation modelling (SEM) to evaluate the effects of the addition of NaCl and SA on ion toxicity, oxidative damage, enzymatic and nonenzymatic antioxidant defences, and the photosynthetic performance and biomass of tobacco plants. Principal component analysis (PCA) was performed, and the corresponding visualizations were generated via Origin 2024 (Origin Lab, USA).

## 3 Results

### 3.1 Effects of salt stress and exogenous SA on plant biomass

The dry weights of the control and salt-stressed tobacco plants, either in the presence or absence of SA, are shown in **Table 1**. It was observed that the biomass of tobacco was significantly reduced by soil salt stress (*p* < 0.05). Compared with those in the CK treatment, the values of SB, RB, and GB in the NaCl treatment decreased by 50.0%, 34.6%, and 46.8%, respectively. In contrast, the application of SA to the control group (CK+SA treatment) resulted in remarkable increases of 16.8%, 9.0%, and 14.9% for SB, RB, and GB, respectively. Under salt stress conditions, the exogenous application of SA significantly increased the biomass of tobacco plants (*p* < 0.05). Specifically, compared with those in the NaCl treatment alone, the SB, RB, and GB contents in the NaCl+SA treatment increased by 74.0%, 35.3%, and 63.5%, respectively. In terms of the R/S ratio, the NaCl treatment significantly increased this ratio, whereas the CK+SA treatment markedly decreased it (*p* < 0.05). However, compared with the CK treatment, the NaCl+SA treatment had no significant effect on the R/S ratio (*p* > 0.05).

**Table 1:** The changes of tobacco dry weight under different NaCl and SA treatments.

Treatment	SB (g)	RB (g)	GB (g)	R/S ratio
CK	2.92 ± 0.02 b	0.78 ± 0.01 b	3.70 ± 0.02 b	0.266 ± 0.004 b
NaCl	1.46 ± 0.02 d	0.51 ± 0.01 d	1.97 ± 0.03 d	0.352 ± 0.004 a
CK+SA	3.41 ± 0.06 a	0.85 ± 0.01 a	4.25 ± 0.06 a	0.249 ± 0.005 c
NaCl+SA	2.54 ± 0.03 c	0.69 ± 0.02 c	3.22 ± 0.04 c	0.270 ± 0.002 b

Values are the mean of four replicates. Different letters in the same column indicate signiﬁcant differences (p < 0.05) between different treatment groups according Duncan’s test.

### 3.2 Effects of salt stress and exogenous SA on Na^+^, K^+^, and MDA levels in tobacco leaves

**Figure 1:**
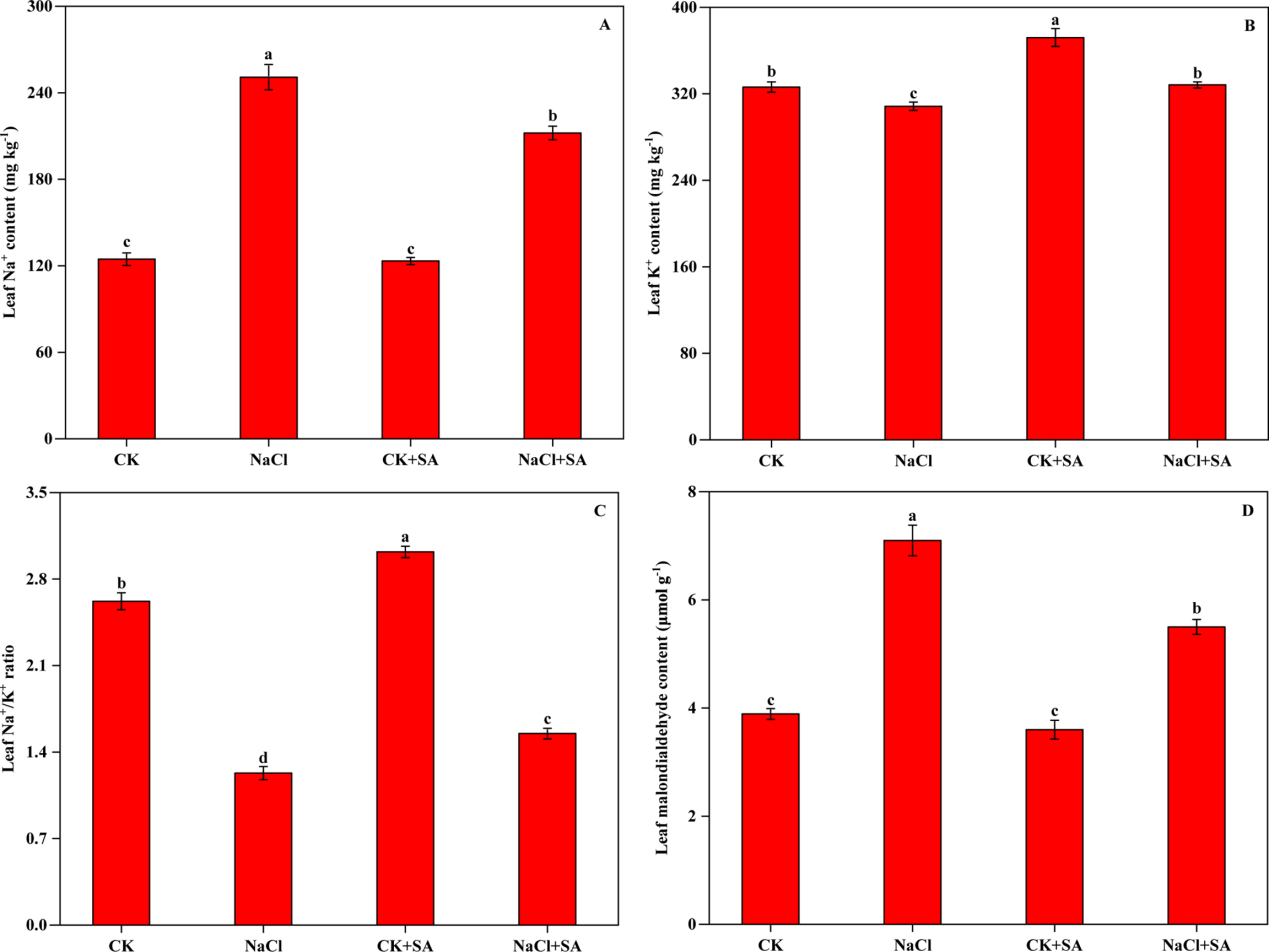
Sodium ion (Na^+^) content **(A)**, potassium ion (K^+^) content **(B)**, K^+^/Na^+^ ratio **(C)** and malondialdehyde (MDA) content **(D)** of tobacco leaves treated with different salt stress and exogenous salicylic acid treatments. Vertical bars represent ± SE of the mean (n = 4); different letters on the SD bars indicate significant differences among different salt stress and exogenous salicylic acid treatments (*p* < 0.05).

The effects of salt stress and exogenous SA on the Na^+^, K^+^, and MDA contents of tobacco plants are depicted in **Figure 1**. Salt stress led to increased Na^+^ accumulation and K^+^ loss, consequently resulting in a significant increase in MDA levels. Compared with those in the control (CK), the Na^+^ and MDA contents in the NaCl treatment increased by 101.3% and 86.8%, respectively, whereas the K^+^ content decreased by 5.4%. Under salt stress conditions, foliar application of SA mitigated these effects by reducing the Na+ and MDA contents and increasing the K^+^ content in tobacco leaves. Specifically, compared with the NaCl treatment alone, the NaCl+SA treatment decreased the Na^+^ and MDA contents by 15.4% and 31.0%, respectively, and increased the K+ content by 6.4%. These alterations in Na+ and K^+^ levels under different salt and SA treatments significantly affected the K^+^/Na^+^ ratio. Compared with that of the control, the K^+^/Na^+^ ratio decreased by 53.1% in the NaCl treatment, increased by 15.3% in the CK+SA treatment, and decreased by 40.8% in the NaCl+SA treatment.

### 3.3 Effects of salt stress and exogenous SA on leaf pigments and chlorophyll a fluorescence

Leaf Chl a, Chl b and Cars levels were strongly inhibited by salt stress. As shown in **Table 2**, compared with those in the CK treatment, the contents of Chl a, Chl b, Chl a+b, and Cars in the NaCl treatment decreased by 1.02, 0.32, 1.32, and 0.27 mg g^-1^, respectively. Furthermore, foliar spray of 1.0 mM SA increased the contents of photosynthetic pigments in tobacco leaves under both normal conditions and salt stress conditions. Compared with those in the NaCl treatment, the Chl a, Chl b, Chl a+b, and Cars contents in the NaCl+SA treatment increased by 36.8%, 20.0%, 31.3%, and 68.8%, respectively. There were no notable differences in the Chl a/b ratio among the CK, NaCl, and CK+SA treatments, but this ratio was significantly greater in the NaCl+SA treatment.

**Table 2:** Effects of NaCl and SA treatments on leaf pigments in tobacco plants.

Treatment	Chl a (mg g^-1^)	Chl b (mg g^-1^)	Chl a+b (mg g^-1^)	Chl a/b	Cars (mg g^-1^)
CK	2.84 ± 0.08 b	0.82 ± 0.03 b	3.65 ± 0.07 b	3.53 ± 0.23 bc	0.62 ± 0.02 b
NaCl	1.82 ± 0.04 d	0.50 ± 0.01 d	2.33 ± 0.05 d	3.67 ± 0.16 b	0.35 ± 0.02 d
CK+SA	3.09 ± 0.06 d	0.95 ± 0.02 a	4.04 ± 0.09 a	3.28 ± 0.02 c	0.72 ± 0.01 a
NaCl+SA	2.49 ± 0.04 c	0.60 ± 0.01 c	3.06 ± 0.05 c	4.17 ± 0.08 a	0.59 ± 0.02 c

Values are the mean of four replicates. Different letters in the same column indicate signiﬁcant differences (p < 0.05) between different treatment groups according Duncan’s test.

Chl a fluorescence induction kinetics were conducted to assess the impact of salt stress and exogenous SA on the photochemical efficiency of photosystem II (PSII) in tobacco leaves (**Supplementary Figure S1** **).** The Chl a induction curves, which reflect the dynamics of PSII photochemistry, indicated significant alterations in response to salt stress and exogenous SA compared with those in the CK. The induction curves for the CK and CK+SA treatments were similar and presented significantly lower fluorescence levels at the O-J and J-I phases than did those of the NaCl and NaCl+SA treatments. As the reaction progressed from the I phase to the P phase, the fluorescence curves for the NaCl, CK+SA, and NaCl+SA treatments increased markedly, surpassing that of the CK treatment.

**Figure 2:**
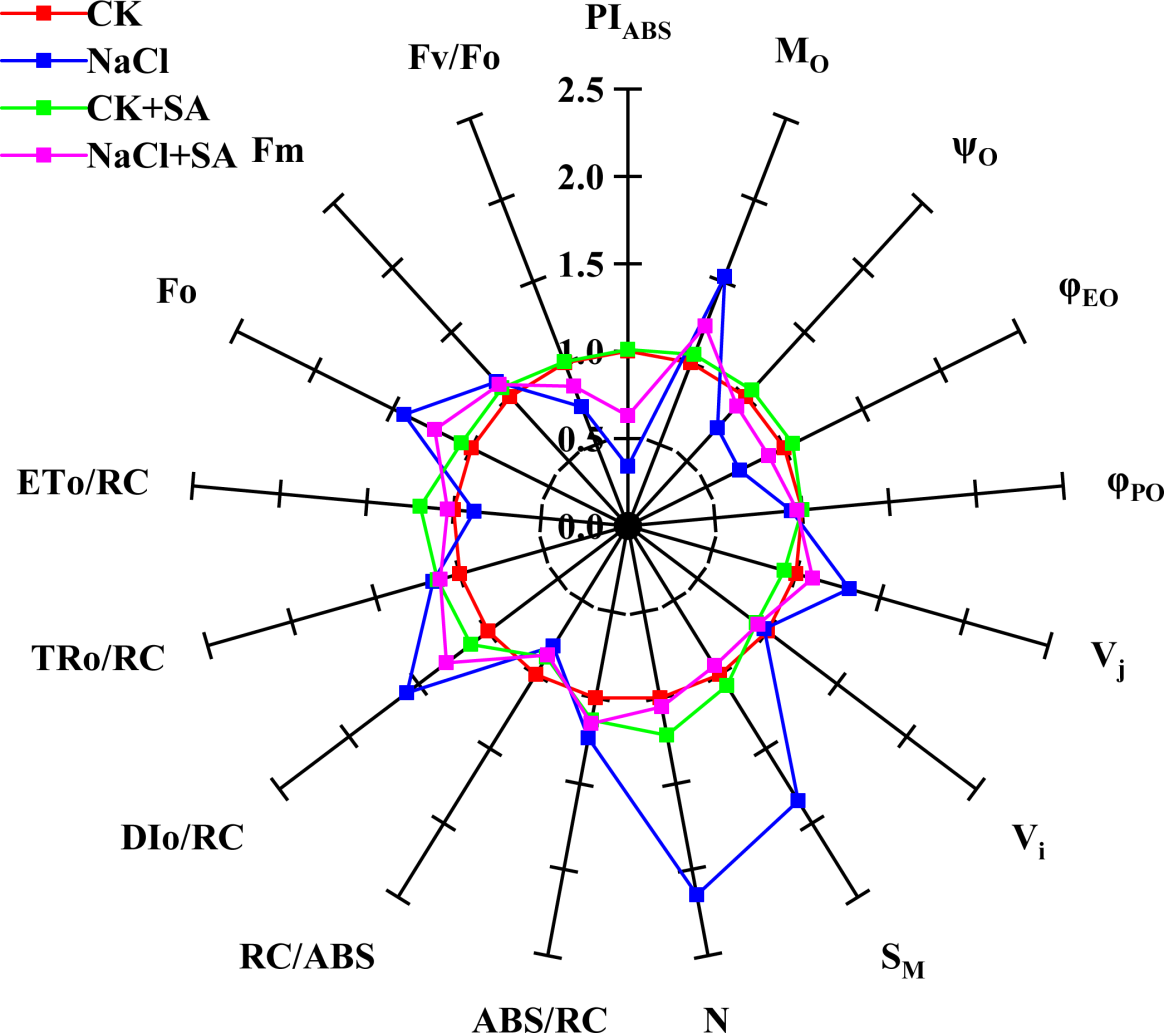
A spider plot’ of selected chlorophyll fluorescence parameters characterizing the photosystem II function of tobacco leaves treated with different salt stress and exogenous salicylic acid treatments. All values are shown as percent of control (control plants=1.0). Fo, the minimal fluorescence; Fm, maximal fluorescence; Fv/Fo, activity of the water-splitting complex; PIABS, performance index; Mo, energy flux rate; ABS/RC, light absorption energy flux per RC; DIo/RC, relative energy flux per PSII RC; ETo/RC, maximum electron transport flux per PSII RC; TRo/RC, trapped energy flux per RC; φ_Po_, maximum quantum yield for primary photochemistry; φ_EO_, quantum yield for electron transport); ψ_o_, probability that an electron moves further than QA; Vi, relative variable fluorescence at the I-step; Vj, relative variable fluorescence at the J-step; S_M_, normalized total complementary area above the OJIP transient; N, number of QA redox turnovers until Fm is reached.

The relative values of the fluorescence parameters, which are crucial for characterizing the functionality of PSII, are graphically represented as a spider plot, as shown in **Figure 2**. This type of plot is particularly useful for visualizing the changes in selected fluorescence parameters relative to a standard or control. The analysis revealed no statistically significant differences in the fluorescence parameters between the control treatment (CK) and the treatment with added exogenous SA (CK+SA treatment). However, under salt stress conditions, notable decreases in the values of PT_ABS_, ψ_O_, φE_O_, RC/ABS, ETo/RC, and Fv/Fo were observed, indicating impaired PSII function. Concurrently, significant increases in Mo, Vj, SM, N, DIo/RC, and Fo were observed. The application of exogenous SA under saline conditions reduced the suppressive effects of salt stress on PSII function. Compared with the NaCl treatment alone, the NaCl+SA treatment significantly reduced the values of Mo, Vj, SM, N, Dio/RC, and Fo, which are indicative of reduced nonphotochemical quenching and improved energy dissipation. Conversely, it increased the values of PTABS, ψO, φEO, RC/ABS, ETo/RC, and Fv/Fo, reflecting a positive impact on the photochemical efficiency and overall functionality of PSII.

### 3.4 Effects of salt stress and exogenous SA on total soluble protein and total phenolic compound contents in leaves

**Figure 3:**
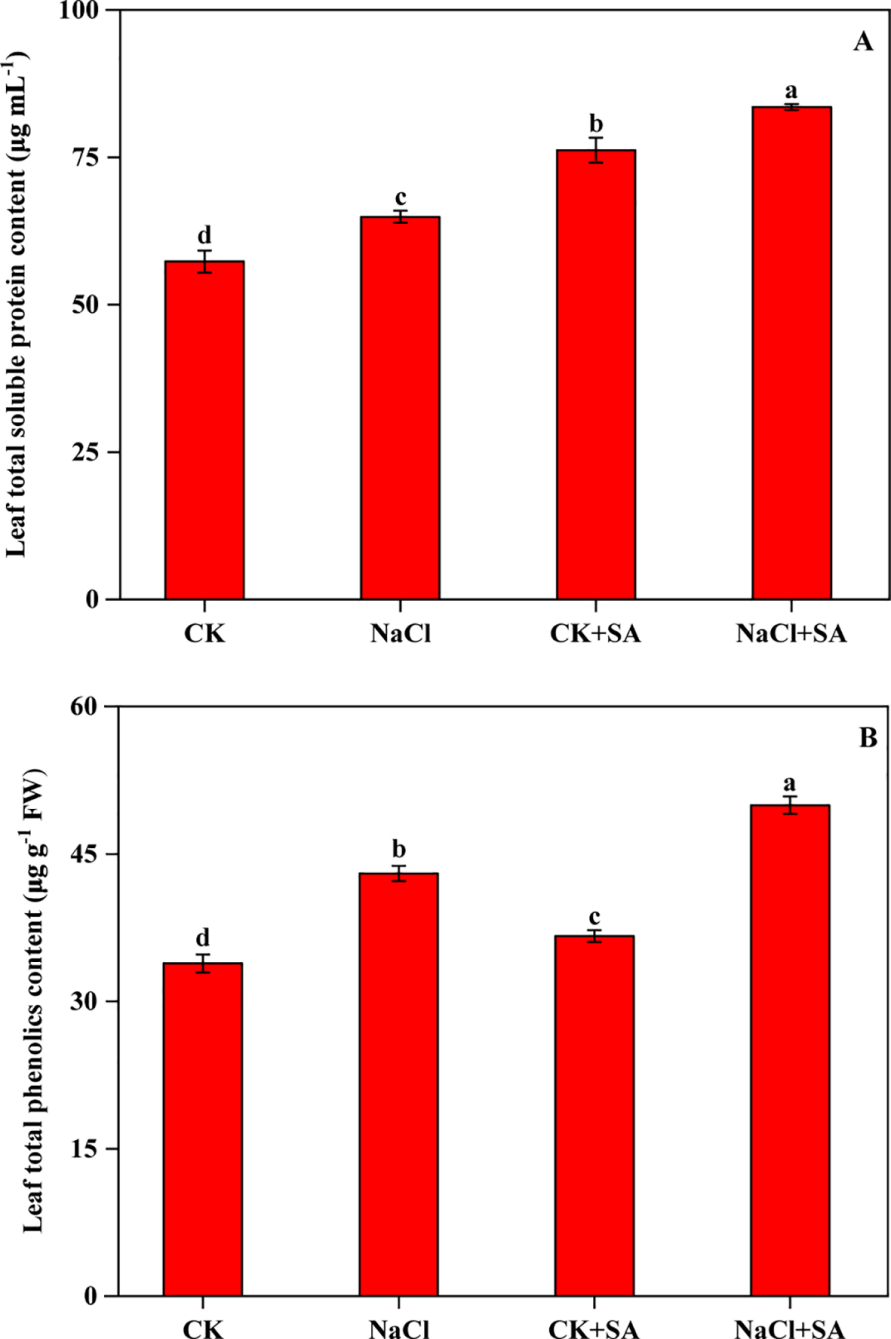
Effects of salt stress and exogenous salicylic acid on the contents of total soluble protein **(A)** and total phenolics **(B)** in leaves of tobacco plants. Vertical bars represent ± SE of the mean (n = 4); different letters on the SD bars indicate significant differences among different salt stress and exogenous salicylic acid treatments (*p* < 0.05).

The variations in TSP and TPC under various salt stress and SA treatments are depicted in **Figure 3**. Both the TSP and TPC levels in tobacco leaves were notably increased in response to salt stress, exogenous SA application, and their combined application. Compared with that in the CK group, the TSP content increased by 13.3%, 33.0%, and 45.7% under the NaCl, CK+SA, and NaCl+SA treatments, respectively. Similarly, the TPC increased by 27.0%, 8.2%, and 47.6% under the same conditions.

### 3.5 Effects of salt stress and exogenous SA on leaf ascorbate and glutathione levels

**Figure 4:**
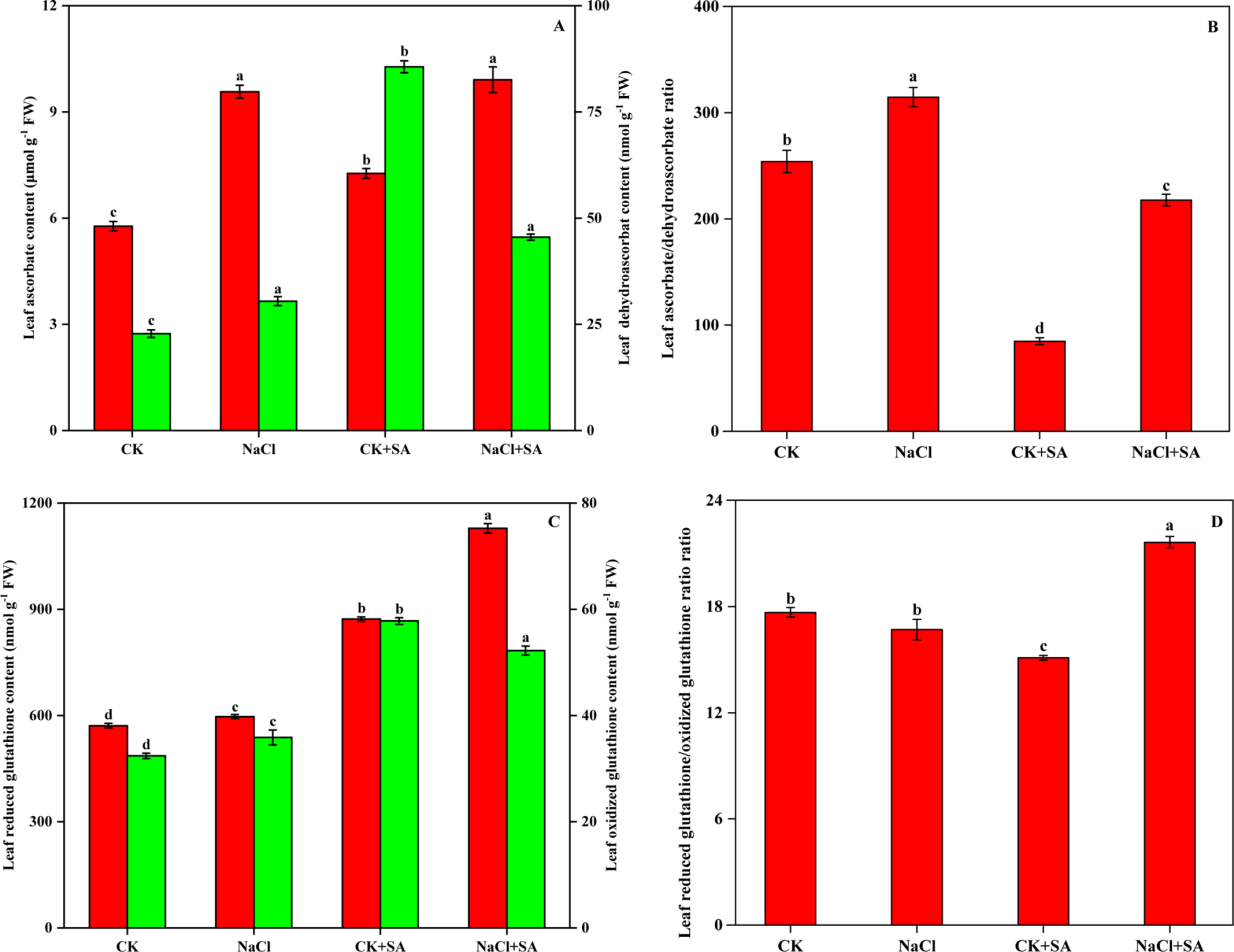
Ascorbate and dehydroascorbate contents **(A)**, Ascorbate/dehydroascorbate ratio **(B)**, Reduced glutathione and oxidized glutathione contents **(C)**, and Reduced glutathione/oxidized glutathione ratio **(D)** in leaves of tobacco treated with different NaCl and SA treatments. Vertical bars represent ± SE of the mean (n = 4); different letters on the SD bars indicate significant differences among different salt stress and exogenous salicylic acid treatments (*p* < 0.05).

The levels of AsA, GSH, and their oxidized counterparts, DHA and GSSG, in tobacco leaves subjected to NaCl and SA treatment are shown in **Figure 4**. Salt stress markedly decreased AsA and GSH levels by 39.7% and 34.5%, respectively. Conversely, DHA and GSSG levels were significantly elevated by 275.9% and 78.5%, respectively. These changes resulted in substantial decreases in the GSH/GSSG and AsA/DHA ratios. In contrast, the addition of exogenous SA resulted in increased levels of AsA and GSH as well as decreased levels of DHA and GSSG. This led to an improvement in the GSH/GSSG and AsA/DHA ratios under both nonstressed and saline stress conditions.

### 3.6 Effects of salt stress and exogenous SA on the activities of ascorbate−glutathione cycle enzymes and defence enzymes

Significant alterations in the activities of key defence enzymes, including SOD, POD, and CAT, were detected across various NaCl and SA treatments, as detailed in **Table 3**. Compared with the control (CK), salt stress notably increased the activities of these enzymes. Specifically, the SOD, POD, and CAT activities increased by 13.8%, 13.8%, and 11.6%, respectively, in the NaCl treatment. Furthermore, under conditions of salt stress, the application of exogenous SA increased the activities of these defence enzymes. Compared with those in the control, the activities of SOD, POD, and CAT were elevated by 18.5%, 18.8%, and 17.7%, respectively, upon NaCl+SA treatment.

**Table 3:** Effects of NaCl and SA on the activities of antioxidant enzyme and the contents of soluble proteins in leaves of tobacco.

Treatment	SOD activity (U g^-1^ protein)	POD activity (U g^-1^ protein)	CAT activity (U g^-1^ protein)	APX activity (U g^-1^ protein)	GR activity (U·mg^-1^ protein)	MDHAR activity (U·mg^-1^ protein)	DHAR activity (U·mg^-1^ protein)
CK	644.4 ± 1.4 c	8667.5 ± 82.0 a	357.1 ± 3.8 c	26.5 ± 0.3 c	6.83 ± 0.03 d	1.29 ± 0.02 b	1.37 ± 0.01 b
NaCl	733.1 ± 73.5 b	9847.8 ± 59.3 b	398.7 ± 6.0 b	35.5 ± 0.5 b	7.88 ± 0.05 c	0.84 ± 0.01 d	0.90 ± 0.03 d
CK+SA	647.2 ± 1.5 c	8727.6 ± 28.7 a	358.5 ± 1.5 c	26.7 ± 0.3 c	8.73 ± 0.04 b	1.35 ± 0.02 a	1.61 ± 0.02 a
NaCl+SA	763.7 ± 1.8 a	10297.9 ± 78.3 b	420.4 ± 1.7 a	40.6 ± 0.3 a	9.58 ± 0.08 a	1.19 ± 0.03 c	1.23 ± 0.05 c

Values are the mean of four replicates. Different letters in the same column indicate signiﬁcant differences (p < 0.05) between different treatment groups according Duncan’s test.

The activities of enzymes associated with the ASA-GSH cycle, namely, APX, GR, MDHAR, and DHAR, were substantially affected by both NaCl treatment alone and in combination with salicylic acid (SA), as presented in **Table 3**. Compared with the CK treatment, the NaCl and NaCl+SA treatments led to significant increases in APX activity (34.0% and 53.2%, respectively) and GR activity (15.4% and 40.1%, respectively). Conversely, NaCl treatment notably suppressed MDHAR and DHAR activity. However, the exogenous application of SA under saline conditions effectively counteracted the inhibitory impact of salt stress on these enzymes. Specifically, the activities of MDHAR and DHAR increased by 41.7% and 36.7%, respectively, in the NaCl+SA treatment compared with those in the NaCl treatment alone.

### 3.7 Effects of salt stress and exogenous SA on gene expression

**Figure 5:**
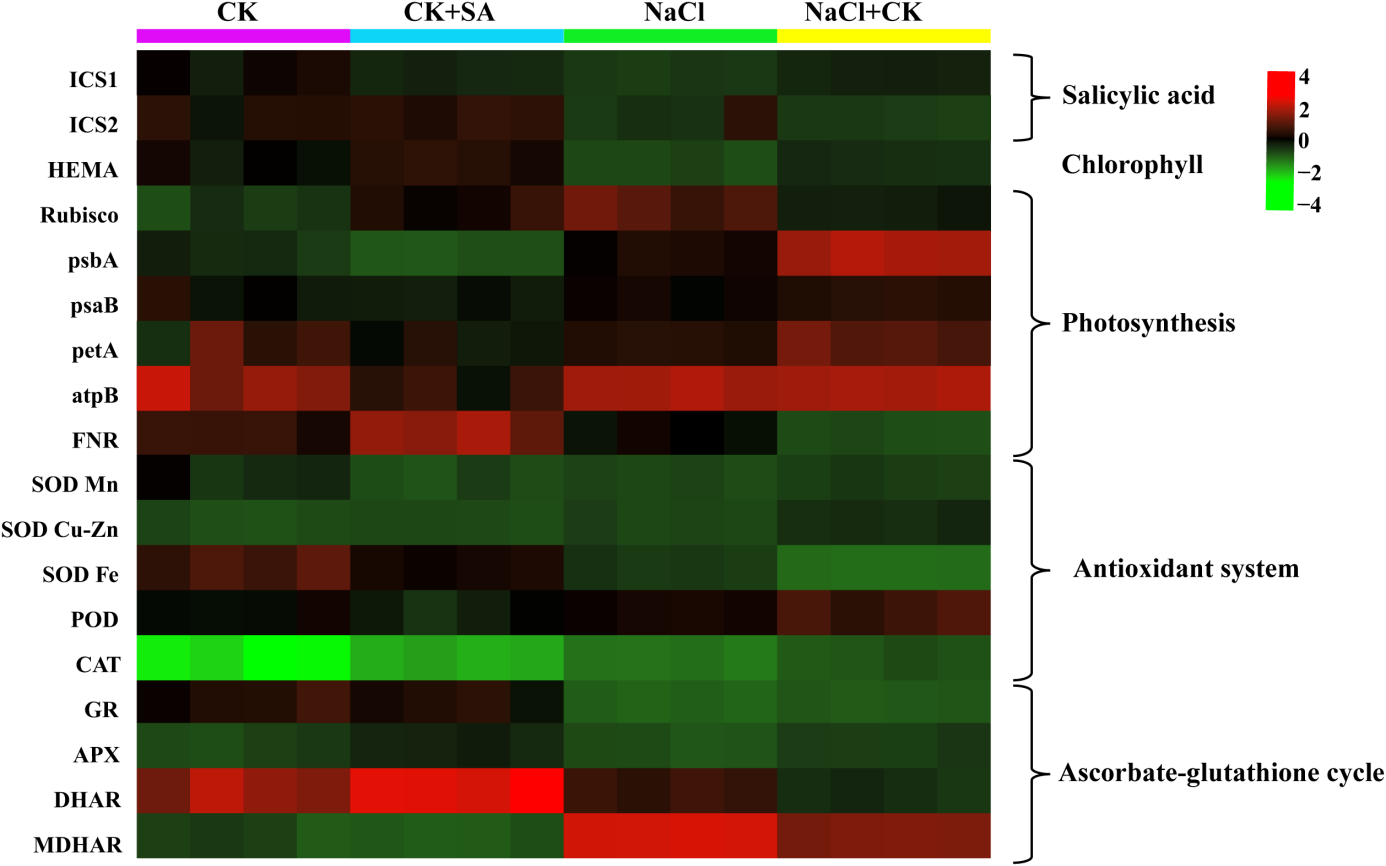
Heatmap showed the influence of SA on the expression levels of salicylic acid, chlorophyll, photosynthesis, antioxidant system, and ascorbate-glutathione cycle related genes in the leaves of tobacco under salt stress.

The transcription levels of genes involved in the biosynthesis of SA and Chl, carbon assimilation, antioxidant systems, and the ascorbate-glutathione (ASA-GSH) cycle, as determined by reverse transcription polymerase chain reaction (RT−PCR) analysis, are presented in **Table 4**. Salt stress markedly reduced the transcript abundance of the SA biosynthesis-related gene *ICS1* by 0.6-fold, but it had no significant effect on *ICS2* (**Table 4**; **Supplementary Figure S2**). The foliar application of SA notably increased the expression levels of both the *ICS1* and *ICS2* genes under both the control and salt stress conditions. NaCl treatment also significantly downregulated the expression of the Chl biosynthesis-related gene *HEMA* and the photosynthesis-related genes *rbcS1*, *pabA*, *psaB*, *petA*, and *FNR* (**Table 4**; **Supplementary Figures S3**, **S4**). These results suggest that leaf pigments, photosynthesis, and the Calvin−Benson−Bassham cycle were severely compromised by salt stress. However, exogenous SA increased the relative expression of these genes 0.1-fold to 0.7-fold under normal conditions and 0.2-fold to 5.6-fold under salt stress conditions. During salt stress, the relative expression of antioxidant system-related genes, including *SOD Fe*, *SOD Cu-Zn*, *SOD M*n, *POD*, and *CAT*, was also significantly modulated (**Table 4**; **Supplementary Figure S5**). Moreover, exogenous SA further strengthened the expression of these genes. With respect to the ASA-GSH cycle-related genes, salt stress markedly altered the expression of APX, MDHAR, and DHAR; however, it did not significantly influence the expression of GR (**Table 4** and **Supplementary Figure S6**). Under salt stress conditions, exogenous SA increased the relative expression levels of *APX*, M*DHAR*, and *DHAR* by 7.3%, 28.0%, and 26.0%, respectively. A heatmap was constructed to visualize the expression levels of all genes under various NaCl and SA treatments (**Figure 5**).

**Table 4:** Relative fold of expression of target genes in different NaCl and SA treatments.

	CK	NaCl	CK+SA	NaCl+SA	*p* value
Salicylic acid					
*ICS 1*	1.74 ± 0.06 c	0.70 ± 0.04 d	2.95 ± 0.10 a	2.14 ± 0.07 b	<0.001
*ICS 2*	1.04 ± 0.02 c	0.86 ± 0.03 c	1.72 ± 0.08 b	2.46 ± 0.15 a	<0.001
Chlorophyll					
*HEMA*	1.39 ± 0.04 c	0.44 ± 0.02 d	2.41 ± 0.08 a	1.59 ± 0.05 b	<0.001
Carbon assimilation					
*rbcS 1*	1.31 ± 0.05 b	0.57 ± 0.02 d	1.80 ± 0.03 a	1.08 ± 0.03 c	<0.001
*psbA*	1.47 ± 0.04 b	0.16 ± 0.01 d	1.66 ± 0.02 a	1.06 ± 0.03 c	<0.001
*psaB*	1.35 ± 0.04 b	0.33 ± 0.01 d	1.67 ± 0.07 a	0.62 ± 0.01 c	<0.001
*petA*	1.01 ± 0.03 b	0.58 ± 0.02 d	1.37 ± 0.02 a	0.80 ± 0.03 c	<0.001
*FNR*	1.22 ± 0.04 b	0.72 ± 0.02 d	1.76 ± 0.05 a	0.88 ± 0.02 c	<0.001
*atpB*	0.46 ± 0.01 b	0.38 ± 0.04 c	0.53 ± 0.01 a	0.43 ± 0.01 bc	0.001
Antioxidant system					
*SOD Fe*	1.11 ± 0.03 c	2.41 ± 0.03 a	1.04 ± 0.02 c	1.77 ± 0.04 b	<0.001
*SOD Cu-Zn*	1.01 ± 0.02 c	2.09 ± 0.3 b	1.02 ± 0.03 c	3.71 ± 0.06 a	<0.001
*SOD Mn*	1.23 ± 0.02 c	1.57 ± 0.09 ab	1.42 ± 0.07 b	1.74 ± 0.02 a	<0.001
*POD*	1.14 ± 0.06 a	0.56 ± 0.02 c	1.10 ± 0.04 a	0.90 ± 0.01 b	<0.001
*CAT*	1.74 ± 0.08 c	2.41 ± 0.05 b	1.79 ± 0.17 c	3.26 ± 0.05 a	<0.001
Ascorbate-glutathione cycle					
*APX*	1.38 ± 0.11 c	1.79 ± 0.09 ab	1.55 ± 0.09 bc	1.92 ± 0.01 a	0.003
*GR*	1.25 ± 0.05 c	1.32 ± 0.03 c	1.44 ± 0.02 b	1.69 ± 0.04 a	<0.001
*MDHAR*	0.98 ± 0.03 b	0.73 ± 0.02 d	1.11 ± 0.01 a	0.92 ± 0.03 c	<0.001
*DHAR*	1.24 ± 0.04 b	0.82 ± 0.02 d	1.36 ± 0.02 a	1.04 ± 0.02 c	<0.001

Values are the mean of four replicates. Different letters in the same row indicate signiﬁcant differences (p < 0.05) between different treatment groups according Duncan’s test.

### 3.8 Comprehensive analysis

**Figure 6:**
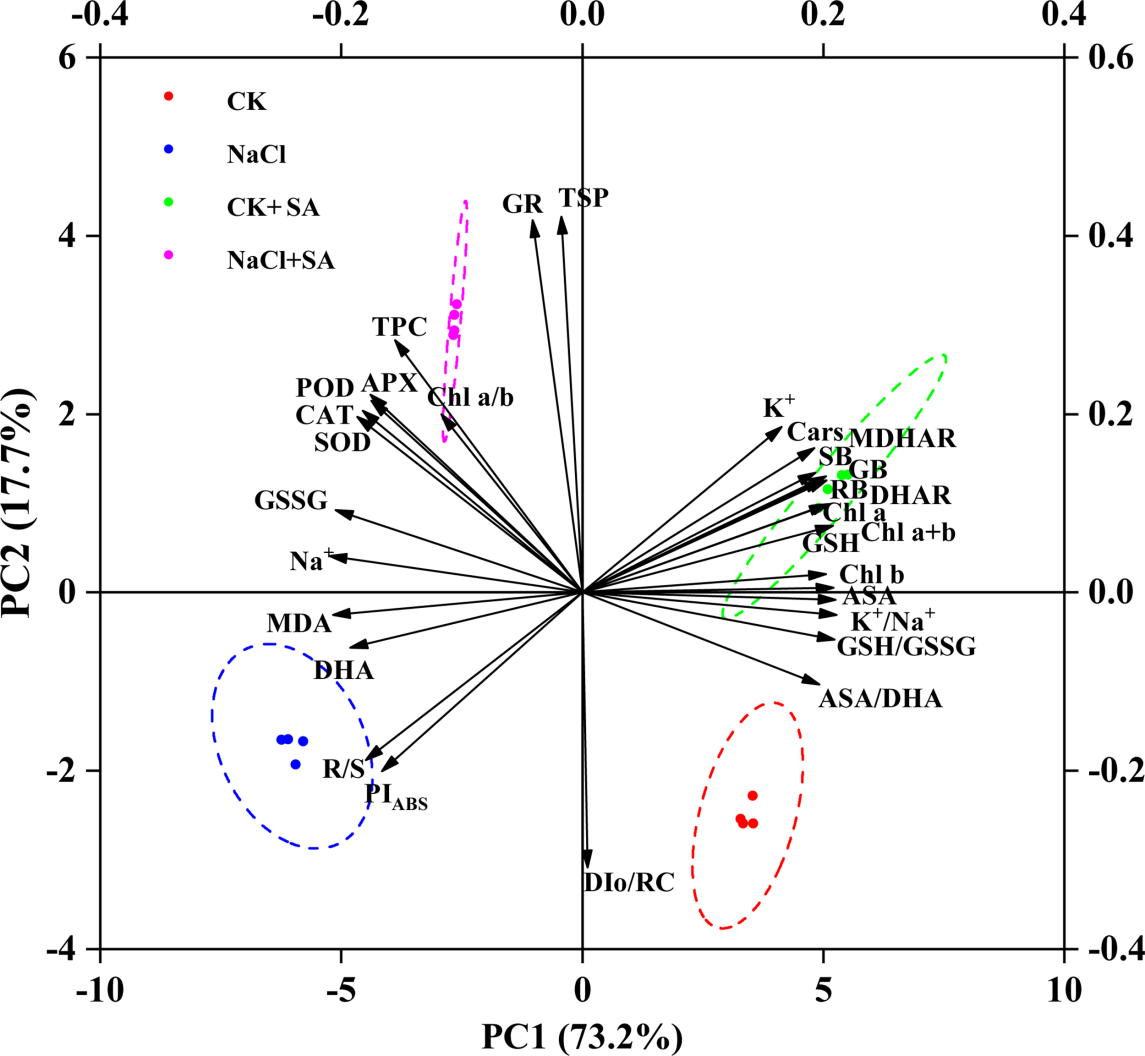
Biplot of first (PC1) and second (PC2) principal components of 30 evaluated traits. APX, Ascorbate peroxidase; ASA, Ascorbate content; ASA/DAH, Ascorbate/dehydroascorbate ratio; CAT, Catalase; Cars, Carotenoids; Chl a, Chlorophyll a; Chl b, Chlorophyll b; Chl a+b- Chlorophyll a+b; Chl a/b, Chlorophyll a/b; DAH, Dehydroascorbate; DHAR, Dehydroascorbate reductase; DIo/RC, Maximum electron transport flux per PSII RC; GB, Gross biomass; GR, Glutathione reductase; GSH, Reduced glutathione; GSH/GSSG, Reduced glutathione/oxidized glutathione ratio; GSSG, Oxidized glutathione content; K^+^, Leaf K^+^; K^+^/Na^+^, leaf K^+^/Na^+^ ratio; MDA, Malondialdehyde; MDHAR, Monodehydroascorbate reductase; Na^+^, Leaf Na^+^; PI_ABS_, Performance index; POD, Peroxidase; RB, Root biomass; R/S, Shoot biomass/root biomass ratio; SB, Shoot biomass; SOD, Superoxide dismutase; TPC, Total phenolics; TSP, Total soluble protein.

To determine the influence of exogenous SA on tobacco plants under salt stress, PCA and correlation analyses were performed on a range of plant physiological and biochemical parameters. As depicted in **Figure 6**, the first two principal components, PC1 and PC2, accounted for 73.2% and 17.7% of the total variation, respectively. The PCA biplot revealed a distinct segregation of the four treatment groups, with high replicability observed among the four replicates per treatment. The variables can be broadly categorized into two clusters. The first cluster, which was proximate to the CK and CK+SA treatments and positioned along the positive axis of PC1, was associated with parameters indicative of plant biomass, photosynthetic efficiency, and antioxidant content. The second cluster, aligned with the positive axis for the NaCl and NaCl+SA treatments, was characterized by markers of Na^+^ toxicity, oxidative stress, and antioxidant system responses. Correlation analysis of various physiological parameters across different NaCl and SA treatments under heat stress conditions (**Supplementary Figure S7**) revealed significant relationships. The Pearson correlation heatmap demonstrated that MDA and Na^+^ were significantly and negatively correlated with Chl a, Chl b, Cars, SB, and RB but were strongly positively correlated with PI_ABS_, TPC, TS, GSH, ASA, SOD, POD, and CAT. Within the ASA−GSH cycle, ASA and GSH were negatively correlated with DHA and GSSG but positively correlated with DHAR and MDHAR. Additionally, soluble sugars were significantly and positively correlated with Chl b, GSH, the GSH/GSSG ratio, K^+^, and the K^+^/Na^+^ ratio.

**Figure 7:**
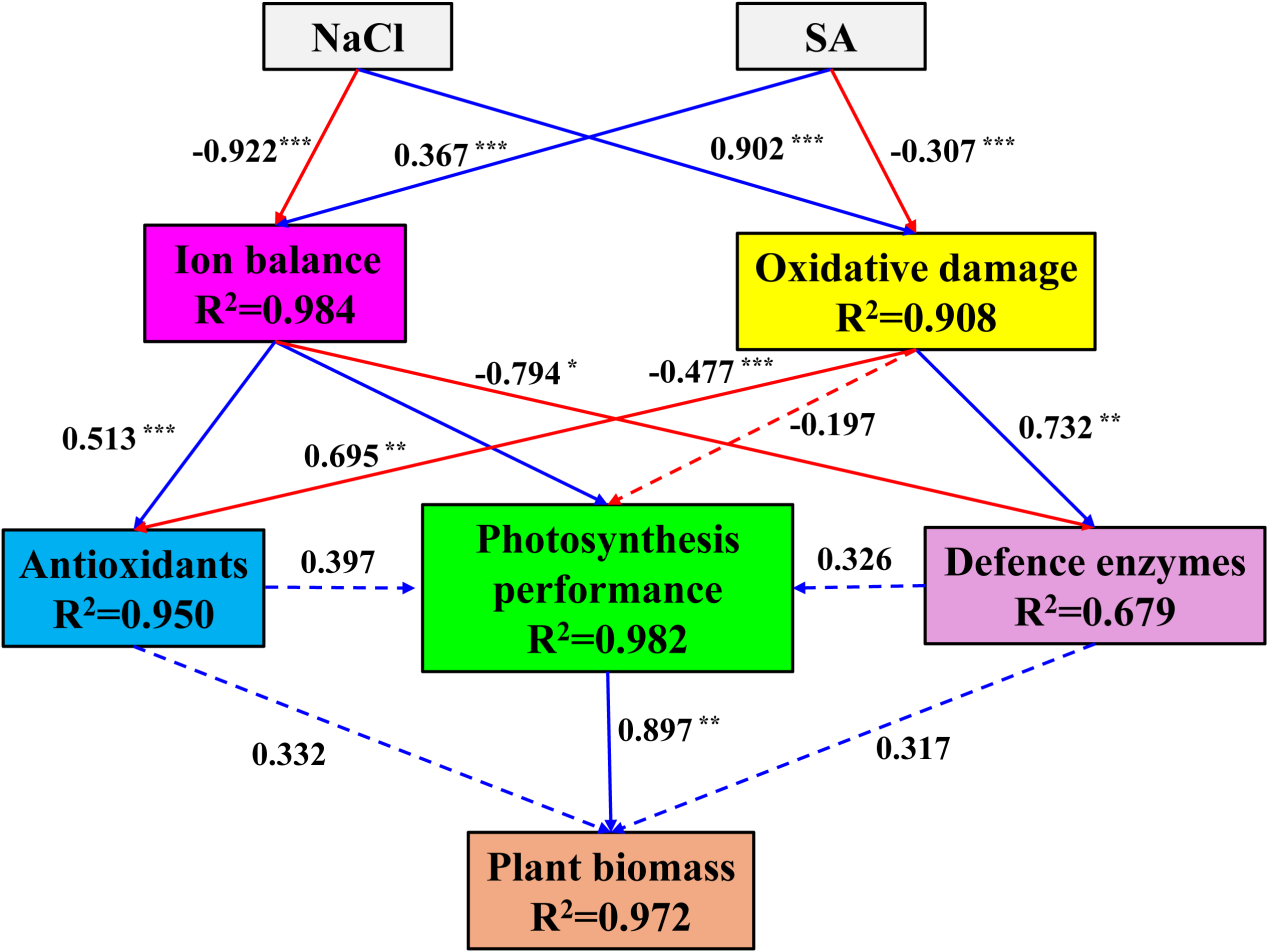
Structural equation model explaining the direct and indirect effects of NaCl and SA on the ion balance, oxidative damage, antioxidant status, photosynthetic capacity, defence enzyme activity, and biomass accumulation in tobacco plants. Standardized path coefficients are shown next to the arrows. Solid line arrows indicate significant path; dotted line arrows indicate not significant paths; blue line arrows indicate positive relationship; red line arrows indicate negative relationship; values associated with line represent standardized path coefficients. The R^2^ numbers within boxes denote the proportion of variance that could be explained by the corresponding variable in the structural equation model. *p<=0.05, **p<=0.01, ***p<=0.001.

To further elucidate the potential effects of NaCl and SA on the ion balance, oxidative damage, antioxidant status, photosynthetic capacity, defence enzyme activity, and biomass accumulation in tobacco plants, SEM was conducted (**Figure 7**). The analysis revealed that both NaCl and SA had significant and direct effects on the ion balance and oxidative damage, albeit with opposing influences. The standardized path coefficients indicated that ion toxicity had a positive and direct effect on antioxidants (path coefficient = 0.513, *p* < 0.001) and photosynthetic performance (path coefficient = 0.695, *p* < 0.01) but a negative and direct effect on defence enzymes (path coefficient = 0.679, p < 0.05). Oxidative damage did not directly affect photosynthetic performance (*p* > 0.05) but did negatively influence antioxidants and positively affect defence enzymes. Neither antioxidants nor defence enzymes had significant direct effects on photosynthetic performance or plant biomass. Moreover, the impact of photosynthetic performance on plant biomass was substantial (path coefficient = 0.897, *p* < 0.01), suggesting that the role of NaCl and SA in determining plant biomass is mediated through their control of ion toxicity on photosynthetic performance.

## 4 Discussion

Soil salinization as a consequence of global climate change and human practices has become an increasing threat to terrestrial ecosystem safety and agricultural sustainability (Palansooriya et al., 2020). Increasing evidence has shown that salt stress negatively impacts plant physiological and biochemical processes, inhibits growth and development, and ultimately reduces yield as a result of osmotic stress from elevated salt content, oxidative stress due to the accumulation of reactive oxygen species, and ionic imbalances stemming from the overabsorption of Na^+^ and Cl^-^ ions (Ahmad et al., 2021; Nxele et al., 2017; Saddiq et al., 2019). The alleviation of soil salinity-induced salt stress represents a significant global challenge in the 21st century (FAO, 2024). As an important signalling molecule, SA has been widely observed to alleviate salt stress and enhance the salinity tolerance of various plant species, such as Asarum sieboldii Miq (Kashif et al., 2024), sunflower (Liu et al., 2024), and Zea mays L. (Barwal et al., 2024). However, the specific mechanisms by which this growth regulator improves plant tolerance to salt stress are still not fully understood. On the basis of these findings, the present study evaluated the beneficial effects of exogenous SA application in alleviating the ion toxicity and oxidative damage caused by NaCl on the growth, physiochemical characteristics, and the expression of key genes in tobacco plants.

### 4.1 Exogenous SA alters the accumulation of Na^+^, K^+^ and MDA in salt-stressed tobacco leaves

Under soil salinity stress, plants absorb an overabundance of Na+ ions into their leaves, disrupting the K^+^/Na^+^ balance across the plasma and thylakoid membranes (Demidchik et al., 2010). This subsequently makes the loss of K^+^ from the leaves unavoidable (Genc et al., 2007) and culminates in toxicity that adversely affects plant processes. In our current study, the content of Na^+^ significantly increased, whereas the K^+^ content substantially decreased in the leaves of tobacco plants subjected to salt stress, resulting in a decreased K^+^/Na^+^ ratio (**Figure 1**). These findings are in line with studies on sweet potato (Kumar et al., 2024) and olive (Methenni et al., 2018) plants. Given the similar physicochemical structures of Na^+^ and K^+^, there is competition by Na^+^ for the binding sites that K^+^ typically occupies (Ahanger and Agarwal, 2017). Under saline conditions, the unassisted entry of Na^+^ across the plasma membrane may cause membrane depolarization, which considerably impedes the uptake of K^+^ and amplifies its loss through channels that are activated by depolarization and facilitate the outwards rectification of K^+^ (Jayakannan et al., 2013). The diminished absorption of K^+^ results in various types of cellular damage and inhibits plant growth because K^+^ is a vital activator of numerous cytosolic enzymes and plays a role in osmotic regulation (Almeida et al., 2017). In contrast, the foliar application of SA is highly effective at reducing the surplus of Na^+^ and increasing the K^+^ content in tobacco plants experiencing salt stress (**Figure 1**). These findings are consistent with studies in which 1.00 mM SA was sprayed on *Egletes viscosa* subjected to 80 mM NaCl (Batista et al., 2019) and *Olea europeae* L. subjected to 200 mmol L^-1^ NaCl (Methenni et al., 2018). Previous studies have reported that SA is capable of diminishing the xylem transport of Na^+^ to the aerial parts of plants and decreasing membrane polarization by stimulating H^+^-ATPase activity and the expression of K^+^-Na^+^ transporters under salt stress, which may subsequently lead to improved Na^+^ retention and reduced Na^+^ efflux (Rubio et al., 2020). Furthermore, maintaining a high K^+^/Na^+^ ratio is recognized as a critical characteristic for plant salinity tolerance (Fariduddin et al., 2018). The increase in the K^+^/Na^+^ ratio caused by exogenous SA under salt stress conditions suggested that SA modifies Na^+^ and K^+^ selectivity which help in lowering membrane damage and maintaining salinity tolerance by regulating ion homeostasis (Isayenkov and Maathuis, 2019; Li et al., 2020).

In addition to soil salinity-induced ionic stress, oxidative damage due to the increased accumulation of ROS is another reason for the decrease in growth of plants exposed to salinity (Liu et al., 2024). Studies have shown that the levels of ROS, such as hydrogen peroxide, singlet oxygen, and hydroxyl radicals, are markedly increased by salt stress (Elshoky et al., 2021). MDA, a by-product of lipid peroxidation (Srivastava et al., 2014), serves as an indicator of cellular membrane damage under salt stress, with increased levels of lipid peroxidation and MDA accumulation reflecting such damage (Kaya et al., 2020). In the present study, a significant increase in the MDA content was observed in salt-stressed tobacco leaves (**Figure 1**), suggesting that such stress promotes MDA accumulation and consequently induces significant oxidative damage to cellular biomolecules (Mahawar et al., 2024). Similar results were reported in studies by Rhaman et al. (2024) and Liu et al. (2024). When the tobacco leaf surface was sprayed with 1.0 mM SA, MDA levels decreased from 7.1 μmol g^−1^ to 5.5 μmol g^−1^ in NaCl-treated plants. These results indicate that SA plays a protective role in effectively diminishing salt-induced ROS accumulation and alleviating membrane oxidation damage in salt-stressed environments.

### 4.2 Effects of exogenous SA on leaf pigments and chlorophyll a fluorescence in salt-stressed tobacco leaves

Chloroplasts in leaves, which are crucial for capturing and conveying light energy in the process of photosynthesis, are particularly vulnerable to abiotic stressors (Polesi et al., 2019). It is well documented that salt stress damages chloroplasts and decreases the Chl content, thereby impeding the capacity for photosynthesis (Chen et al., 2021). In the present study, Chl a, Chl b and Cars concentrations were markedly reduced in salt-stressed tobacco plants (**Table 2**), indicating that salt stress strongly inhibited the biosynthesis of leaf pigments and accelerated their degradation. Similar results were also reported in studies by Ashraf and Harris (2013) and Nieves-Cordones et al. (2019). The decreased leaf pigment contents could be attributed to the accumulation of Na+, the depletion of K^+^, and the subsequent lipid peroxidation within the leaf cells of tobacco plants (Nongpiur et al., 2016; Taïbi et al., 2016). Earlier studies demonstrated that the use of growth regulators can significantly reduce Chl breakdown and increase the salt tolerance of plant seedlings (Sardar et al., 2023). In our research, Chl a and Chl b concentrations were substantially greater in plants treated with NaCl+SA than in those treated with NaCl alone, suggesting that foliar SA application promotes pigment biosynthesis and slows pigment degradation under salt stress, possibly because of decreased oxidative stress (Farhangi-Abriz and Ghassemi-Golezani, 2018). In addition to the leaf Chl content, the Car content was also noticeably increased by SA in salt-stressed leaves (**Table 2**). Previous studies have revealed that Cars are essential for safeguarding photosynthesis from light damage and play a significant role as signalling molecules in plant development, especially when plants are subjected to various abiotic and biotic stresses (Ashraf and Harris, 2013). The increased Car content suggested that spraying with exogenous SA strengthened the plant’s self-protection ability and salt tolerance by initiating an array of metabolic and physiological processes (Hafeez et al., 2024).

Elevated Na^+^ levels lead to iron-induced toxicity and oxidative stress, which not only damage chloroplasts but also adversely impact the structure and function of the photosynthetic apparatus, consequently increasing ROS levels (Pan et al., 2021). Among the photochemical processes, PSII is the most vulnerable to the disruptive effects of NaCl stress, largely due to the toxicity of Na^+^ ions (Jajoo, 2013). To evaluate PSII activity in plants treated with different levels of NaCl and SA, OJIP transients, which are indicative of the photosynthetic electron transport rate and the efficacy of light energy capture by the PSII reaction centre, were employed in our study. The ascent from the O to J step in the OJIP transient curves is known as the photochemical phase (Stirbet and Govindjee, 2011). Our results showed that salt stress led to a more rapid increase in fluorescence during the O−J phase (**Supplementary Figure S1**). These results suggest that the reoxidation of QA^−^ is impeded and that there is an increased accumulation of QA^−^ due to the diminished efficiency of electron transport past QA (Kalaji et al., 2016). Soil salinity resulted in remarkable increases in Fo, Fv/Fo and PI_ABS_ (**Figure 2**). These findings are consistent with the findings of Che et al. (2022) and Li et al. (2020). The increase in Fo pointed to salt stress-induced inhibition of the PSII reaction centre, which restricted electron transfer from QA to QB and lowered the efficacy of energy trapping within PSII. Fo is associated with the D1 protein, which is a key component of PSII. The deregulation of *PsbA* expression also suggested that the D1 protein was suppressed by salt stress. The Fv/Fo parameter represents the performance of the water-splitting complex on the donor side of PSII (Schreiber et al., 1995). A diminished Fv/Fo ratio signifies that salt stress significantly compromised the photosynthetic electron transport chain. This could be due to the inhibition of osmotic water uptake under stress (Fricke and Peters, 2002) and the disruption of the electron transport and photophosphorylation processes, which can hinder ATP synthesis (Pereira et al., 2000). Furthermore, the inhibited expression of photosynthetic electron transport-related genes such as *FNR* in our study suggested that electron transport was prevented by salt stress. PI_ABS_, an indicator of photosynthetic efficiency, represents the potential for energy conservation by the photosynthetic machinery following the absorption of photons by PSII and the subsequent reduction in intersystem electron acceptors (Strasser et al., 2004). The decreased PI_ABS_ in the salt stress treatment group revealed a diminished efficiency in the number of functional reaction centres and the ability to channel and convey electrons to the electron transport chain (Kalaji et al., 2011). With the application of 1.0 mM SA, the values of Fo, Fv/Fo and PI_ABS_ markedly increased (**Figure 2**), and the expression of carbon assimilation-related genes, including *rbcS 1*, *psbA*, *psaB*, *petA*, *FNR*, and *atpB*, significantly increased (**Table 4**; **Figure 5**). These results suggested that the detrimental effects of salt stress on tobacco leaf photosynthesis performance were effectively mitigated by SA. This amelioration may be attributed to the beneficial effects of SA on repairing the damaged photosynthetic apparatus, such as the oxygen-releasing complex and PSII reaction centre, promoting the efficiency of electron transfer between PSII and PSI through the cytochrome b6f complex, increasing F-type ATPase activity, and promoting Rubisco synthesis. These results are consistent with those of Ahmad et al. (2018), who reported that SA effectively increases photosynthetic electron transport, sustains higher Rubisco activities, and enhances PSII efficiency in the leaves of *Vicia faba* L. plants grown under 100 mM salt stress conditions. Lotfi et al. (2020) reported that the application of 1.0 mM SA enhanced the I−P phase of the OJIP transient, elevated the PI_ABS_ index, and optimized energy management for reaction centre closure, thereby reducing the impact of salinity stress on PSII in mung bean plants exposed to salt stress ranging from 3 to 9 dS/m^2^.

### 4.3 Effects of exogenous SA on leaf antioxidant contents in salt-stressed tobacco leaves

Phenolic compounds, among the most significant secondary metabolites in plants, serve as nonenzymatic defence strategies for quenching ROS and act as biochemical indicators of a plant’s adaptation to environmental stress (Boudet, 2007; Khalil et al., 2018). Our present study revealed that the TPC in tobacco leaves was significantly increased by the addition of NaCl and that this increase was strengthened by the foliar application of SA (**Figure 3**). The increased TPC in salt-stressed tobacco leaves reflects the self-defence mechanism by which the plants avoid oxidative damage (Saini et al., 2024). The increase in TPC under salt stress conditions due to the application of SA can be attributed to the increased activities of enzymes such as phenylalanine ammonia-lyase, which are involved in the biosynthetic pathways of phenolic compounds (Chaman et al., 2003; Sánchez-Rodríguez et al., 2011). SA is a phenolic compound in plants, and the expression levels of the isochorismate pathway genes *ICS1* and *ICS2*, which are crucial for SA biosynthesis, are strongly correlated with SA accumulation (Ding and Ding, 2020; Wildermuth et al., 2001). In our research, the transcription of ICS1 and ICS2 in tobacco leaves was considerably elevated with NaCl+SA treatment compared with NaCl treatment alone (**Table 4** and **Figure 5**). These results suggest that SA supplementation promotes the production of phenolic compounds in response to salt stress. The exogenous application of SA increases the accumulation of phenylpropanoids, thereby increasing phenolic acid levels in *Vitis vinifera* L (Chen et al., 2006). Gohari et al. (2024) reported that the addition of SA significantly enhanced phenolic compounds, including chlorogenic acid, aringin, o-coumaric acid and catechin hydrate, in the leaves of *Valerianella locusta* L. under saline conditions. SS levels also increased in salt-stressed tobacco leaves. These results are consistent with studies related to tobacco (Hu et al., 2024) and *Capsicum annuum* L (Barwal et al., 2023). but contrast with studies focused on *Mentha spicata* L (Shiri et al., 2023). and *Calendula officinalis* L (Baniasadi et al., 2018). under salt stress conditions. The accumulation of TSPs in plants under salt stress correlates with the breakdown of specific structural proteins and a shift in the proteomic profile, aiming to bolster cellular osmotic pressure regulation, neutralize surplus ROS, and preserve enzyme stability (Kaushal et al., 2011; Ozturk et al., 2021). Interestingly, regardless of whether the SS levels were reduced or increased by salt stress, all the above mentioned studies unanimously revealed that the contents of TSPs were markedly increased by the foliar application of SA (Shiri et al., 2023). The increased protein levels following the application of exogenous SA indicate that this protectant facilitates the controlled degradation of proteins by controlling the activity of enzymes such as glutamine synthetase and nitrate reductase, which represent key players in protein synthesis (Misra and Saxena, 2009; Nigam et al., 2022).

### 4.4 Modulation of AsA–GSH cycle activity by exogenous SA in salt-stressed tobacco leaves

The ASA−GSH cycle is acknowledged as the paramount nonenzymatic antioxidant defence system essential for plant resistance to environmental stressors (Nazar et al., 2015). GSH and AsA constitute the majority of soluble antioxidants within the plant system (Foyer and Noctor, 2011); play pivotal roles in the AsA–GSH cycle for destroying photosynthetically generated H_2_O_2_, scavenging lipid peroxides, detoxifying xenobiotics; and maintain the cellular redox status to favour better adaptation to environmental stresses in plants (Noctor et al., 2018; Ramzan et al., 2023). In our study, salt stress led to a notable reduction in the reduced forms of GSH and AsA, whereas their oxidized forms, GSSG and DHA, substantially increased in tobacco plants, resulting in lower AsA/DHA and GSH/GSSG ratios (**Figure 4**). These findings suggest that salt stress disrupts the cellular redox equilibrium by influencing the AsA and GSH pools, potentially affecting cellular functions (Raja et al., 2020). The addition of exogenous SA helped to re-establish the redox balance of the AsA and GSH pools, as evidenced by elevated AsA/DHA and GSH/GSSG ratios in plants treated with NaCl+SA (**Figure 4**). This positive effect of SA on redox balance is similar to that observed in salt-stressed Chinese cabbage (Kaya et al., 2020) and drought-stressed tomato plants (Kaya et al., 2023). APX, GR, MDHAR, and DHAR enzymes are central to the regulation of AsA−GSH cycle metabolism, and our findings revealed that salt stress significantly increased the activities of these four enzymes in tobacco plants (**Table 3**), which aligns with previous findings in mustard plants (Zaid et al., 2019). The exogenous application of SA in our research increased the activities of enzymes related to the AsA−GSH cycle in salt-stressed pepper plants, echoing the results of Kaya et al. (2020). SA enhances AsA−GSH cycle-related enzymes by modulating the transcript levels of antioxidant genes, a mechanism that improves plant salt tolerance by upregulating genes such as GSH, MDHAR, glutathione-S-transferase, GR, DHAR, and GPX, as reported by Jayakannan et al. (2013) and Li et al. (2013). Our research also revealed that the transcription levels of the genes encoding APX, GR, MDHAR, and DHAR were markedly increased upon foliar application of SA (**Table 4**; **Figure 5**). APX is a pivotal enzyme within the AsA-GSH cycle, facilitating the detoxification of H_2_O_2_ to H_2_O by using AsA as an electron donor (Leng et al., 2021). The upregulation of the *APX* gene expression indicates that exogenous SA is advantageous for the specific function of APX in preserving cellular redox balance, thereby enhancing the plant’s ROS scavenging capacity and bolstering its resistance to oxidative stress. GR preserves the regeneration state of GSH, which can defend the photo-protection against oxidative stress or ROS production (Gill et al., 2013). The overexpression of *GR* gene has been shown to mitigate oxidative stress and modulate cellular redox homeostasis in salt-stressed tobacco seedlings (Melchiorre et al., 2009). In the AsA-GSH cycle, MDHAR catalyses the conversion of MDHA into AsA, and DHAR reduces DHA to AsA utilizing GSH as a hydrogen donor (Hossain et al., 2018). Under saline stress conditions, the activities of MDHAR and DHAR, along with the relative expression levels of their corresponding genes in tobacco leaves, were enhanced by the application of exogenous SA, contributing to the regeneration of AsA and the accumulation of GSH (Zhu et al., 2022). These findings indicate that the exogenous application of SA represents an efficient strategy for regulating the AsA−GSH cycle by increasing the activity of antioxidant enzymes and their gene expression in tobacco plants under saline stress, thereby countering the oxidative damage induced by ROS.

### 4.5 Regulation of the nonenzymatic antioxidant system by exogenous SA in salt-stressed tobacco leaves

In addition to the nonenzymatic antioxidant system, which is influenced primarily by the AsA−GSH cycle, the enzymatic antioxidant system is another defence strategy in plants against salinity stress by scavenging excessive ROS (Mahawar and Shekhawat, 2019; Mahawar et al., 2024). The antioxidant defence system comprises enzymes, including SOD, POD, CAT, and APX, which are responsible for ROS scavenging. SOD is typically recognized as the primary antioxidant enzyme that dismutates superoxide radicals into H_2_O_2_, a compound subsequently neutralized into H_2_O and O_2_ by CAT, POD, and APX (Khalvandi et al., 2021; Nabilah et al., 2016). Our research revealed increased SOD, CAT, POD, and APX activities in tobacco plants under NaCl stress (**Table 3**), corroborating studies on various plant species, including tomato (Faizan et al., 2021), hybrid P*ennisetum* (Li et al., 2020), and rice (Koc et al., 2024). The application of SA to NaCl-stressed plants led to a significant increase in the activities of these enzymes, suggesting that SA strengthens the redox defence mechanism against salt stress. This finding is consistent with research highlighting the role of SA in increasing antioxidant enzyme activity and shielding plants from oxidative stress (Fan et al., 2022; Faried et al., 2017). Real-time PCR experiments indicated that the activities of SOD, CAT, POD, and APX in tobacco plants treated with SA are regulated at the genetic level. Compared with nontreated plants, SA-treated tobacco leaves under salt stress presented increased expression of *the SOD-Fe*, *SOD-Cu-Zn*, *SOD-Mn*, *CAT*, *POD*, and *APX* genes (**Table 4**; **Figure 5**). The upregulation of these genes paralleled the increase in enzyme activity, indicating that SA application stimulates the biosynthesis of antioxidant enzymes by modulating gene transcription, which in turn enhances salt tolerance and antioxidant effects (Wang et al., 2016). These results are consistent with those of El-Esawi et al. (2017) and Zhang et al. (2024), who reported that the activities and gene expression of antioxidant enzymes induced by salt stress were substantially alleviated by the application of exogenous SA.

### 4.6 Comprehensive analysis

To further assess the potential influences of NaCl and SA on the growth of tobacco plants, PCA and SEM were conducted. PCA is a well-established, unsupervised multivariate statistical technique that has been widely used to distinguish the differences in controlled treatments and cluster experimental traits with similar characteristics (Li et al., 2023). In our study, the PC results clearly separated the different NaCl and SA treatments into four groups (**Figure 6**). The NaCl group was highly positively related to Na^+^, MDA, DHA, and GSSG and negatively related to plant biomass, leaf pigments, K^+^ and photosynthetic efficiency. In contrast, the other parameters, such as SOD, POD, CAT, APX, TPC and TSP, were significantly and positively correlated with the NaCl+SA group. These findings revealed that NaCl caused severe oxidative stress and iron toxicity during tobacco growth and that exogenous SA can trigger redox defence mechanisms to prevent oxidative damage by regulating antioxidant defence systems and maintaining osmotic balance. SEM was employed to identify the direct and indirect influences of oxidative damage, ion balance, enzymatic antioxidants, and nonenzymatic antioxidants on the photosynthetic capacity and biomass of tobacco plants. According to the findings of the structural equation model, NaCl had a direct effect on the germination of oxidative damage (path coefficient = 0.902, *p* < 0.01) and alterations in the ion balance path coefficient = -0.922, *p* < 0.01) in tobacco plants (**Figure 7**). SA had a significant positive effect on the ion balance (path coefficient = 0.367, *p* < 0.01) and a direct negative effect on oxidative damage (path coefficient = -0.307, *p* < 0.01), suggesting that the foliar application of SA alleviated salt-induced phytotoxicity by maintaining the balance between Na^+^ and K^+^ and alleviated oxidative damage. Furthermore, the ion balance has a direct and significant effect on photosynthesis performance, whereas the influences of oxidative damage, antioxidants, and defence enzymes on photosynthesis performance are not noticeable. As photosynthesis performance was highly related to tobacco biomass (path coefficient = 0.897, *p* < 0.01), we inferred that the regulation of ion homeostasis by SA plays a determinant role in the enhancement of photosynthetic efficiency and biomass in salt-stressed tobacco plants.

## 5 Conclusion

In our study, 1.0 mM SA was sprayed onto the leaves of tobacco plants subject to salt stress to determine the efficacy of this exogenous hormone in mitigating the detrimental effects of salt stress. This intervention led to a reduction in the sodium ion concentration, curbed membrane lipid peroxidation, facilitated potassium ion absorption, preserved the cellular osmotic balance, increased pigment levels in leaves, increased the photosynthetic capacity, and consequently improved the overall biomass of tobacco. Furthermore, AsA−GSH cycle metabolism, the activities of antioxidant enzymes, and the concentrations of nonenzymatic antioxidants such as phenolic compounds (TPCs) and total phenolic compounds (TSPs) were markedly elevated in response to SA supplementation, which increased the expression of specific genes. These findings showed that foliar application of SA can substantially bolster the salt tolerance of tobacco plants by modulating the transcriptional activity of genes associated with SA and Chl biosynthesis, carbon fixation, and both enzymatic and nonenzymatic antioxidant mechanisms, thus strengthening the plant’s defences against oxidative stress under saline soil conditions. The use of SA potentially represents an ecologically sound and economically feasible solution for the management of saline soils and the reclamation of marginal and wastelands that are currently underutilized.

## Data Availability

The original contributions presented in the study are included in the article/**Supplementary Material**. Further inquiries can be directed to the corresponding authors.
