# Supplementary material for: Physiological and molecular mechanisms of exogenous salicylic acid in enhancing salt tolerance in tobacco seedlings by regulating antioxidant defence system and gene expression
Source: Front Plant Sci. 2025 Jan 31;16:1545865. doi: 10.3389/fpls.2025.1545865 (PMC11825763; doi:10.3389/fpls.2025.1545865)
Supplement: Supplementary file 1 [file DataSheet1.docx]

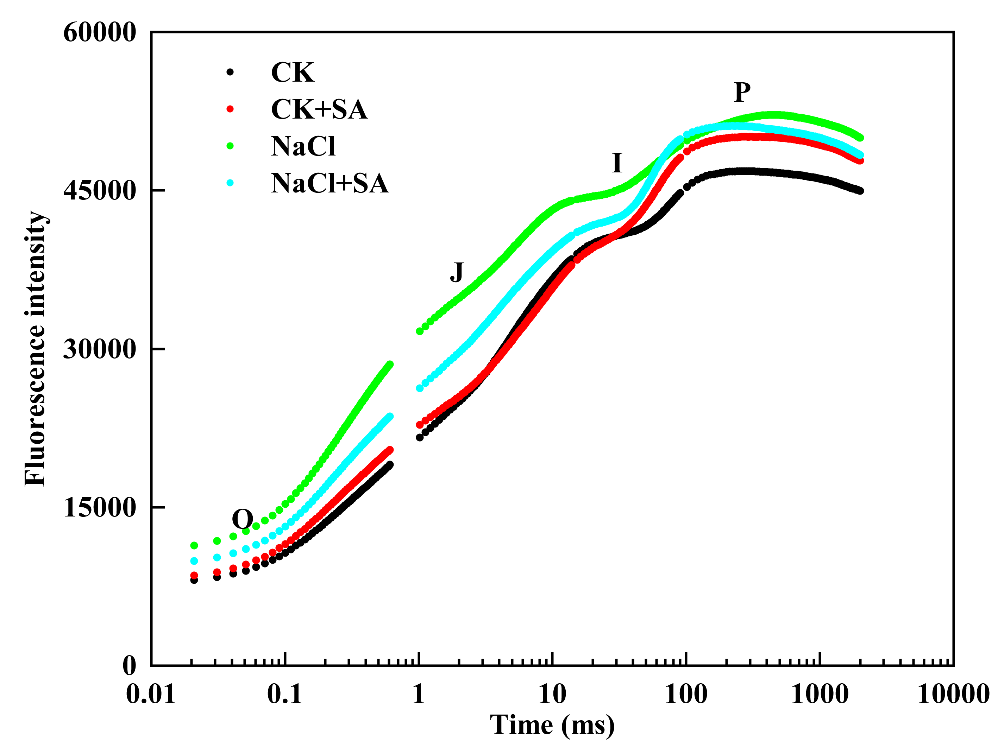


**Figure S1** The ﬂuorescence intensity of the original ﬂuorescence kinetic curve of tobacco leaves under different NaCl and SA treatments.


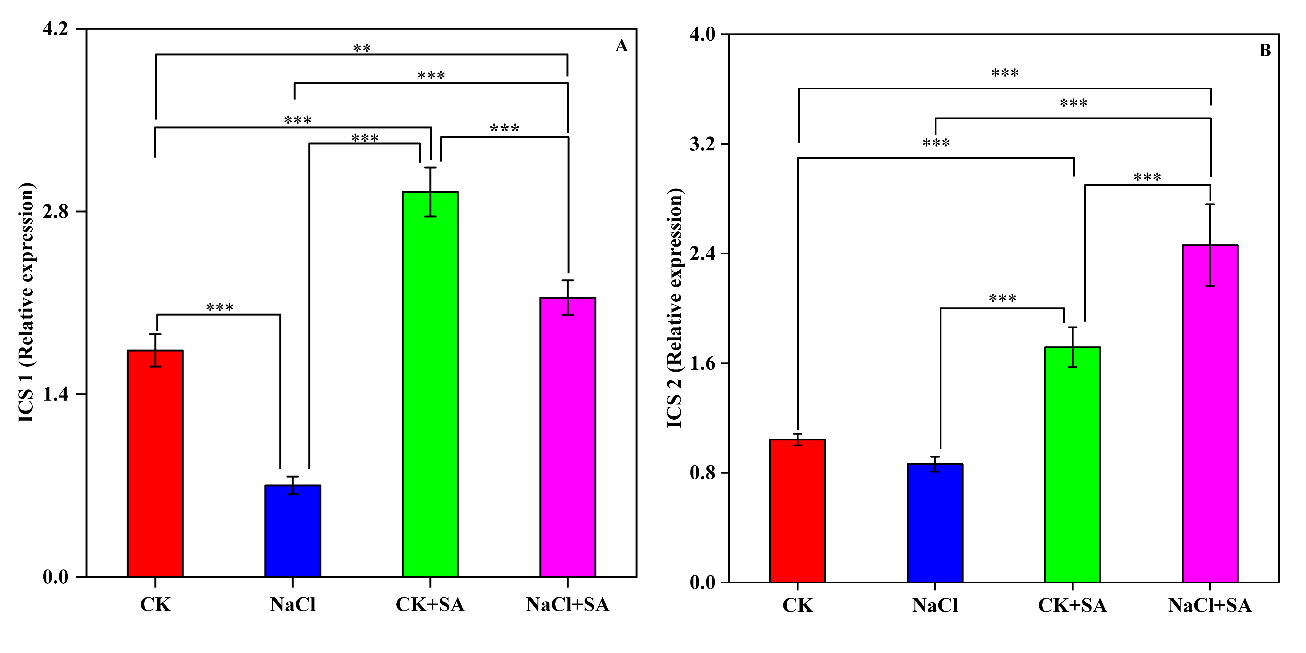


**Figure S2** Relative expression of *ICS1* (**A**) and *ICS2* (**B**) genes in different NaCl and SA treated tobacco leaves.


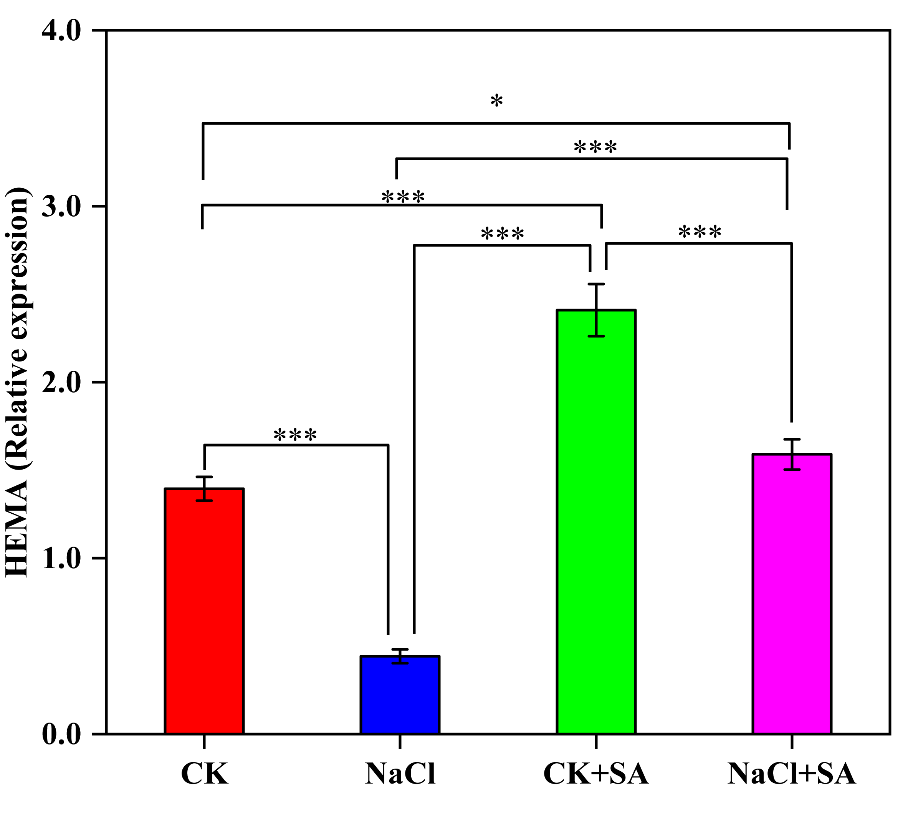


**Figure S3** Relative expression of *HEMA* gene in different NaCl and SA treated tobacco leaves.


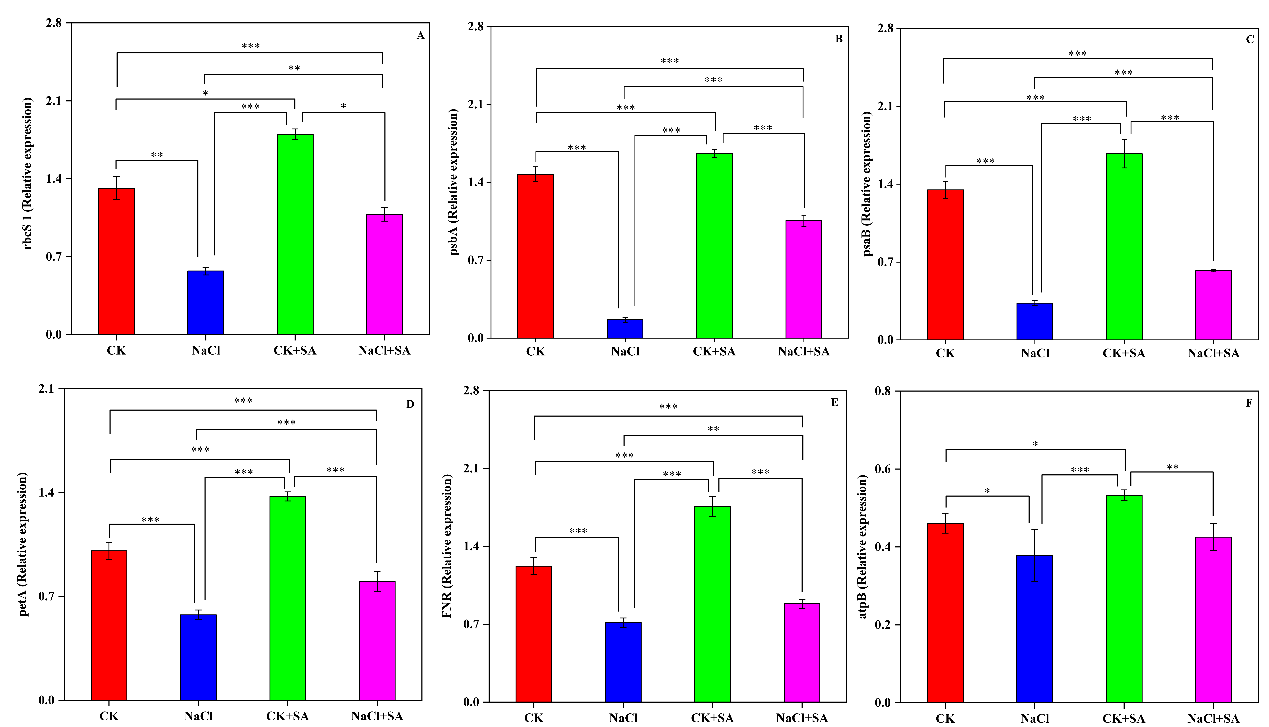


**Figure S4** Relative expression of *rbcs1* (**A**), *psbA* (**B**), *psaB* (**C**), *petA* (**D**), *FNR* (**E**), and *atpB* (**F**) genes in different NaCl and SA treated tobacco leaves.


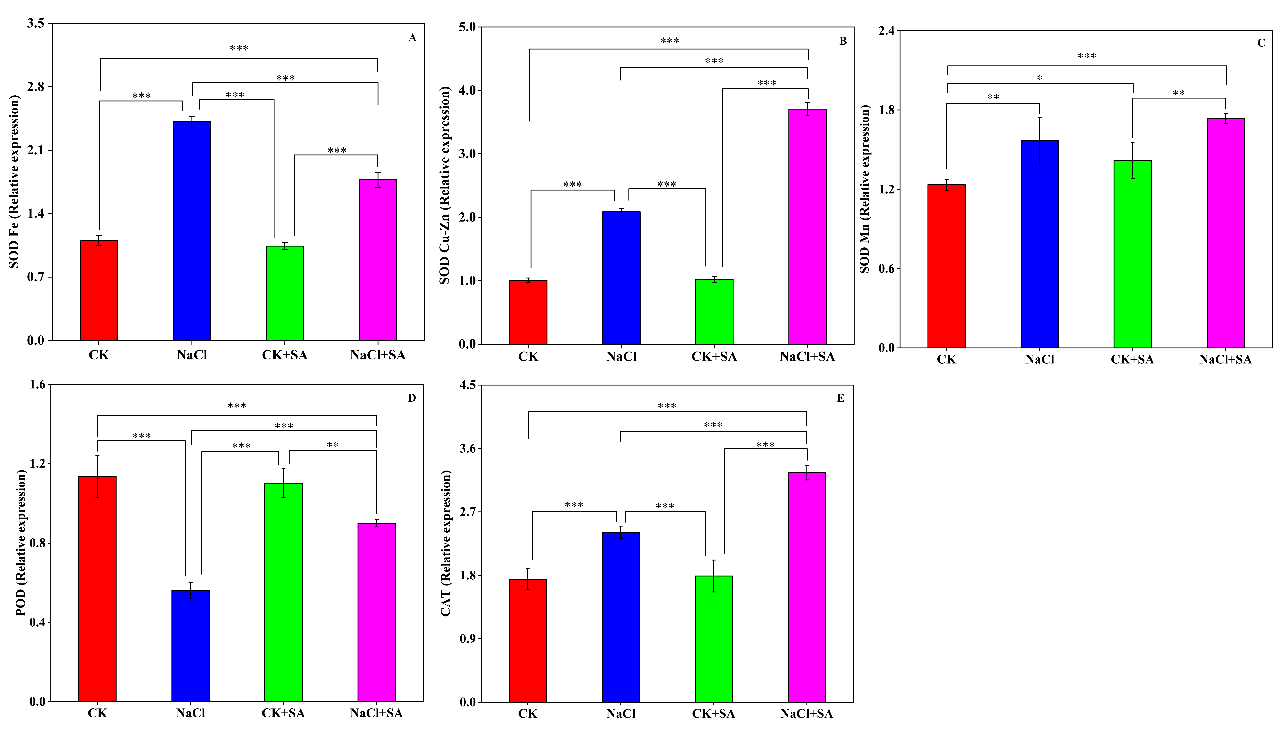


**Figure S5** Relative expression of *SOD Fe* (**A**), *SOD Cu-Zn* (**B**), *SOD Mn* (**C**), *POD* (**D**), and *CAT* (**E**) genes in different NaCl and SA treated tobacco leaves.


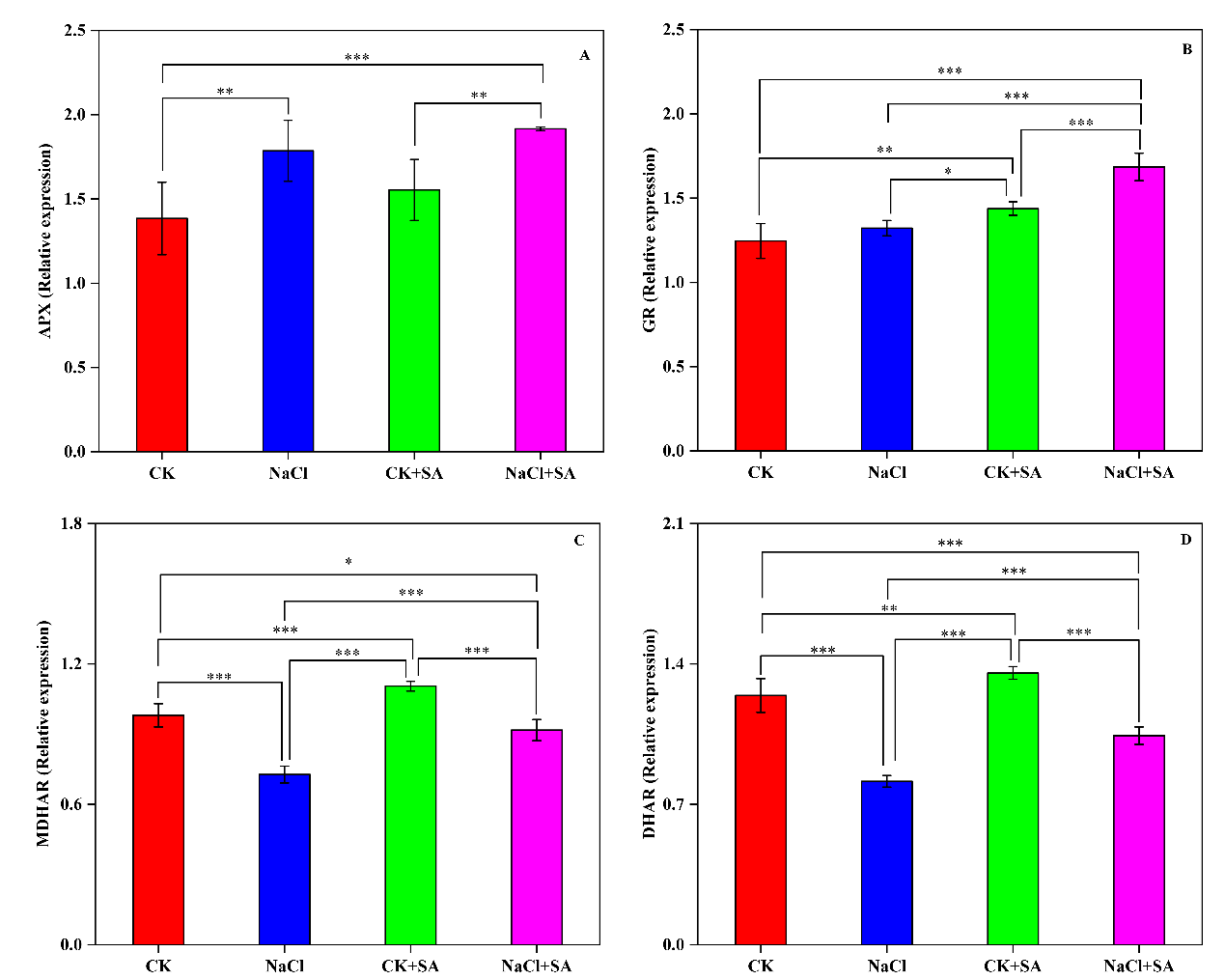
**Figure S6** Relative expression of *APX* (**A**), *GR* (**B**), *MDHAR* (**C**), and *DHAR* (**D**) genes in different NaCl and SA treated tobacco leaves.


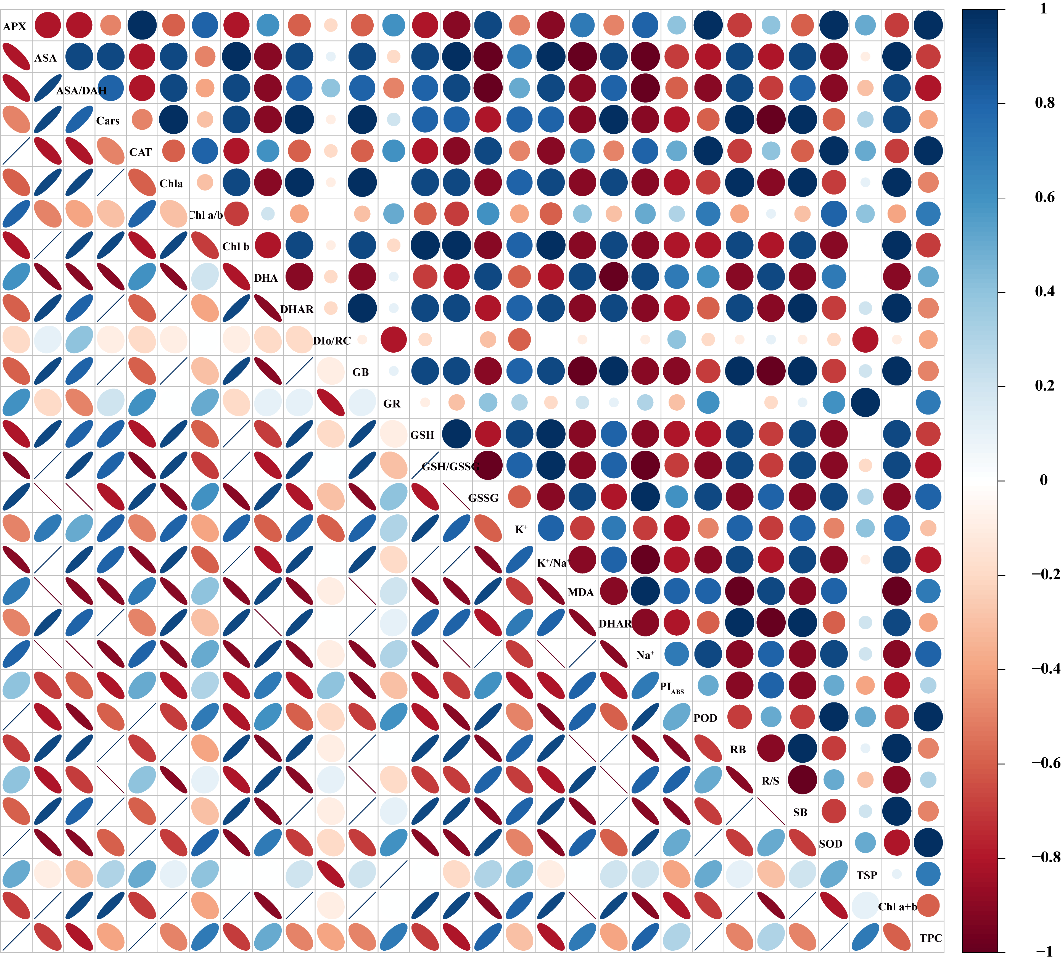


**Figure S7** Heatmap based on the Spearman correlation matrix of different plant physiological and biochemical parameters in different NaCl and SA treatments. APX- Ascorbate peroxidase; ASA- Ascorbate content; ASA/DAH- Ascorbate/dehydroascorbate ratio; CAT- Catalase; Cars- Carotenoids; Chl a- Chlorophyll a, Chl b- Chlorophyll b, Chl a+b- Chlorophyll a+b, Chl a/b- Chlorophyll a/b; DAH- Dehydroascorbate; DHAR- Dehydroascorbate reductase; DIo/RC- Maximum electron transport flux per PSII RC; GB-Gross biomass; GR- Glutathione reductase; GSH- Reduced glutathione; GSH/GSSG- Reduced glutathione/oxidized glutathione ratio; GSSG- Oxidized glutathione content; K^+^- Leaf K^+^; K^+^/Na^+^- leaf K^+^/Na^+^ ratio; MDA- Malondialdehyde; MDHAR- Monodehydroascorbate reductase;Na^+^- Leaf Na^+^; PI_ABS_- Performance index; POD- Peroxidase; RB-Root biomass; R/S- Shoot biomass/root biomass ratio; SB- Shoot biomass, SOD- Superoxide dismutase; TPC- Total phenolics; TSP- Total soluble protein.

**Table S1** qRT-PCR primer sequence of differentially expressed genes in transgenic alfalfa under salt stress.

| Gene name | Forward primer (5′-3′) | Reverse primer (3′-5′) |
| --- | --- | --- |
| *actin* | CTGAGGTCCTTTTCCAACCA | TACCCGGGAACATGGTAGAG |
| *ICS 1* | GCTTTCCCATCGTTTCTTGTAC | CTTGGCACCCATTCATTGAC |
| *ICS 2* | GCTAATTTCCTTTATCTCTCCC | AATGAACAAGACTGATGATATCT |
| *HEMA* | CTAAAGGAGGTTGTGGCAGCTA | GGGACCATGAAGGAGCTTGTTA |
| *rbcS 1* | AGCTCGTAATGAAGGACGTGA | CAAAACGTCCACTGCTGCAA |
| *psbA* | CGCATACCCAGACGGAAACT | TTACCCAATCTGGGAAGCGG |
| *psaB* | TTTGTGGATGGAATCCCCCG | CCAAGGGTTAGGCCCACTTT |
| *petA* | CCCGTGGAGATTGAGGTTCC | TAAGAACAGCCCCCACGTTC |
| *FNR* | CGCCTTTTTCGTTTGTTTGCTC | CGCCTTTTTCGTTTGTTTGCTC |
| *atpB* | CACCAACGATCCGAGGTTGA | CCTGCTCCTGCTACGACATT |
| *SOD Fe* | CTCCTCCTTATCCCATGGATGC | AAGTTGTCGACATAAGCCCTGT |
| *SOD Cu-Zn* | TGGTCCAAACTCAGTTGTTGGA | CCAGTGGTAAGGCTGAGTTCAT |
| *SOD Mn* | AAGTTGAAACCTCACACTCGGT | GGTTCGAAGTGCCATTTGGAAA |
| *POD* | TGTCATTAGCTAAGCCACGGTT | TGATGGATCAGAACCTCCTCCA |
| *CAT* | GACCCCAGAGGATTTGCTGTAA | ACCATGTCAGGGAACTTCATCC |
| *APX* | TCCGCCCATTGGTTGAGAAATA | CGGACATGGCAAAAGCTACAAA |
| *GR* | AAGCAGTCCATCTGAGGGAAAG | CTCACAAGCACCTTTAAAGCCC |
| *MDHAR* | AGGGTCAAGTTGTTGGGTCTTT | CCATTACAAGAGTGGAACCCGA |
| *DHAR* | AGGTGGCTCTTGGCCATTTC | AACTTCCTGCGAAACAACGG |
